# Structure and potential role of T6SS effector PdpC in *Francisella tularensis* intracellular lifestyle

**DOI:** 10.1038/s42003-026-10543-y

**Published:** 2026-06-25

**Authors:** Xiaoyu Liu, Daniel L. Clemens, Bai-Yu Lee, Xian Xia, Hongcheng Fan, Kaelyn Y. Feng, Marcus A. Horwitz, Z. Hong Zhou

**Affiliations:** 1 School of Life Sciences, Central China Normal University, Wuhan, China; 2 Department of Microbiology, Immunology and Molecular Genetics, University of California, Los Angeles (UCLA), Los Angeles, CA USA; 3 The California NanoSystems Institute (CNSI), UCLA, Los Angeles, CA USA; 4 Department of Medicine, University of California, Los Angeles (UCLA), Los Angeles, CA USA; 5 State Key Laboratory of Virology and Biosafety, College of Life Sciences, TaiKang Center for Life and Medical Sciences, Wuhan University, Wuhan, Hubei China

**Keywords:** Supramolecular assembly, Infection

## Abstract

*Francisella tularensis*, a gram-negative facultative intracellular pathogen, causes the often-fatal disease tularemia. Its *Francisella* pathogenicity island (FPI) encodes a type VI secretion system (T6SS) critical for virulence, yet the mechanisms by which T6SS effectors enable intracellular replication remain poorly understood. Here, we report the structure of the T6SS effector PdpC, revealing a novel protein fold with no identifiable homologs. The structure resembles a seahorse, comprising five domains: an N-terminal domain, a central body domain (CBD), a wedge domain, a C-terminal tail, and an unmodeled “mouth” domain. Biochemical analyses demonstrate that PdpC binds host phospholipids, particularly phosphatidylinositol-3-phosphate, and interacts with PdpE, another FPI-encoded protein. Structure-guided functional studies show that the CBD alone suffices for these interactions, while PdpE is secreted via a T6SS-independent pathway. These findings explain how the sophisticated effector duo enables phagosomal escape and establishes infection: PdpC is delivered by T6SS to PI(3)P-enriched early phagosomal membranes to rupture phagosomes to escape, and then coordinates with PdpE to promote intracytoplasmic *Francisella* replication. This work transforms PdpC from an orphan virulence factor into a tractable molecular switch at the heart of *Francisella*’s intracellular lifestyle and a potential target for anti-tularemia therapeutics.

## Abstract (web summary)

Structural and functional analyses identify PdpC as a *Francisella* T6SS effector that selectively binds phospholipids and interacts with PdpE, suggesting an effector duo coordinating phagosomal escape and intracellular replication.

## Introduction

F*rancisella tularensis* (Ft) subsp. *tularensis* is a gram-negative facultative intracellular bacterial pathogen that causes a serious and potentially fatal infection, tularemia, in humans^1,2^. Because of its high infectivity, with doses as low as 25 colony-forming units capable of causing severe morbidity and mortality, Ft subsp. *tularensis* is considered an agent of bioterrorism^1^. Macrophages are the primary host cells for *F. tularensis* in animals^3^. After uptake by macrophages via the novel mechanism of looping phagocytosis^4^, *F. tularensis* initially resides in a phagosome that resists fusion with lysosomes and does not acquire acid hydrolases, such as cathepsin D^5^. Within hours, the bacterium escapes from its phagosome and multiplies extensively in the macrophage cytosol^6^.

**Fig. 1:**
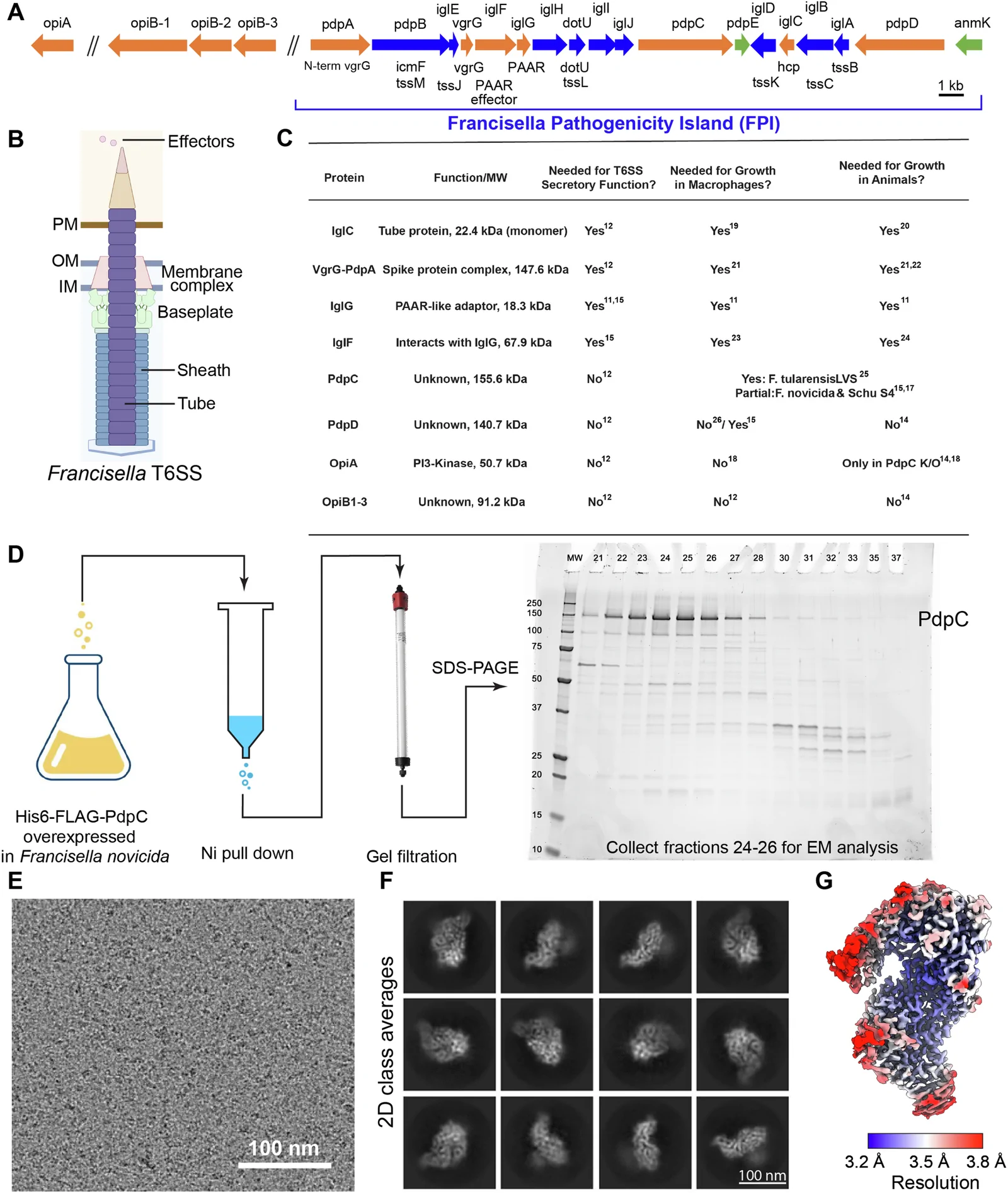
Structure determination of T6SS effector Protein PdpC*.* **A** T6SS related genes including T6SS gene cluster on the FPI. Genes encoding proteins that have been shown to be secreted by the *F. novicida* T6SS are shown in orange. Genes encoding known structural components of the T6SS (sheath, baseplate, membrane complex) are shown in blue. The genes encoding PdpE and AnmK, which have not been demonstrated to be secreted by the T6SS are shown in green. Canonical orthologs, where known, are shown at the bottom. **B** Schematic of *Francisella* T6SS. IM, OM, and PM refer to the inner membrane, outer membrane, and phagosomal membrane, respectively. **C** *Francisella* proteins secreted via the T6SS apparatus. **D** Protein sample preparation flowchart and the SDS-PAGE gel of PdpC. **E** CryoEM micrograph of PdpC. **F** Representative 2D class averages of PdpC particles. **G** CryoEM reconstruction of PdpC colored by local resolution.

*F. novicida* (Fn) (also known as *F. tularensis* subsp. *novicida*) is often used as a model for Ft subsp. *tularensis* because it is genetically closely related and exhibits the same intracellular lifestyle but is less virulent to humans. Within the *F. tularensis* genome, the *Francisella* pathogenicity island (FPI) is essential to the capacity of both Ft and Fn to escape from their phagosome, replicate inside macrophages, and cause fatal disease in animals^7,8^. Whereas Ft has two copies of its FPI, Fn has only a single copy, thereby facilitating genetic manipulation. The FPI encodes a type VI secretion system (T6SS) that secretes effector molecules that act on the target cell. Several proteins encoded within the FPI (Fig. 1A) have been shown to be secreted in a T6SS-dependent fashion (Fig. 1B)^9^, including IglC, VgrG, PdpA, IglF, IglG, PdpC, and PdpD^10–13^. Four additional proteins encoded outside of the FPI (OpiA and OpiB1-OpiB3) also have been shown to be secreted in a T6SS-dependent fashion^12^. AnmK, encoded within the FPI, is thought to be an anhydro-N-acetylmuramic acid kinase, but its secretion has not been demonstrated and its role in pathogenesis is unclear. It is also unclear whether VgrG, PdpA, IglC, IglF, and IglG, which are essential for T6SS-mediated protein secretion, solely serve as structural components secreted alongside effectors or additionally exhibit effector functions in infected host cells. On the other hand, disruption of genes encoding PdpC, PdpD, OpiA, or OpiB1-OpiB3 does not impact secretion of other T6SS proteins^12,14^, supporting their role as secreted effector proteins. Deletion of the *pdpC* gene in *F. novicida* has been shown to cause a severe defect in phagosome escape and a partial defect in virulence in mice^15^. Deletion of both *pdpC* and *pdpD* causes an even more profound defect in *F. novicida* phagosome escape in macrophages and combined deletion of *pdpC*, *pdpD*, and *anmK* renders *F. novicida* avirulent in a mouse model^15^. In the *Galleria mellonella* (i.e., wax moth) larva model, the presence of an individual gene of *pdpC*, *pdpD*, or *opiA* alone, together with functional T6SS secretory capacity, is sufficient for full virulence^14^. In the SCHU strain of *F. tularensis* subsp. *tularensis*, PdpC has been shown to be essential to the capacity of the bacterium to grow in macrophages and cause disease in mice^16^. An intermediate phenotype with decreased phagosome escape in macrophages and attenuated virulence in mice has been shown for the Schu S4 strain with deletion of both *pdpC* genes^17^. While OpiA has been demonstrated to be a PI3-kinase that delays phagosome maturation^18^, the biological functions of other secreted effector proteins (Fig. 1C), including PdpC, remain largely unknown^11,12,14,15,17–26^.

The current study aims to elucidate the structure and function of PdpC, which is one of the key effector proteins encoded by the FPI and secreted via the T6SS. Although PdpC has been implicated in phagosomal escape and virulence, its molecular architecture and mechanistic role remain poorly understood. By cryogenic electron microscopy (cryoEM), we determined the atomic structure of recombinantly produced *Francisella* PdpC at 3.4 Å resolution, revealing a seahorse-like monomeric architecture composed of five domains. We further investigated its biochemical properties and found that PdpC binds selectively to host phospholipids, including phosphatidylinositol-3-phosphate [PI(3)P], and that it binds to another FPI encoded protein, PdpE. We expressed the coding sequence of the central body domain (CBD) of PdpC and found that this domain possesses both PI(3)P and PdpE binding activity. These findings establish a structural and functional framework for understanding the role of PdpC in *Francisella* pathogenesis.

## Results

### CryoEM reveals a seahorse-shaped architecture of PdpC from *F. novicida*

PdpC is a key effector protein secreted by the *Francisella* type VI secretion system (T6SS) (Fig. 1B). During purification of His_6_-FLAG-tagged PdpC from *F. novicida*, we observed co-purification of FPM13 (FTN_1118), a periplasmic metal-binding protein with similar size and affinity for nickel resin^27^. To obtain homogeneous PdpC for structural characterization, we produced the tagged protein in a ΔFPM13 background with supplementation of 5% KCl^10^ to induce T6SS formation. Size-exclusion chromatography yielded a dominant ~150 kDa species corresponding to PdpC (Fig. 1D), which was collected and used for subsequent cryoEM analysis.

In our initial cryoEM analysis of PdpC purified from *F. novicida*, only donut-shaped particles were observed, which were later identified as the FPM13 complex^27^. When PdpC was synthesized in the ΔFPM13 *F. novicida* strain, the donut-shaped particles were no longer observed; however, no discernible particles could be seen on Quantifoil holey grids under the microscope. Extensive optimization was performed to overcome this, including adjusting the protein buffer composition, testing various grid types, and adding detergents to alter the properties of the air-water interface. Ultimately, graphene oxide (GO)-coated grids enabled cryoEM imaging and reconstruction of PdpC reported in this study. CryoEM imaging and 2D classification revealed comma-shaped particles (Fig. 1E, F). Due to the low signal-to-noise ratio and severe preferred orientation of the particles, we employed spIsoNet^28^ during data processing to improve 3D reconstruction quality. This approach yielded a 3.4 Å resolution map (Fig. 1G, Supplementary Fig. S1A–C), which supported atomic model building.

PdpC is a 156 kD protein consisting of 1325 amino acids. The cryoEM structure reveals that PdpC exists as a monomer in solution and adopts an asymmetric elongated shape, like a seahorse (Fig. 1G). No homologous structures of PdpC have been reported to our knowledge. Notably, the AlphaFold prediction of PdpC displays very low confidence and differs markedly from our experimentally determined cryoEM structure (Supplementary Fig. S1D), underscoring the novelty of this fold and the difficulty of de novo model building. We used Model-Angelo^29^ to generate a starting model, which was then manually rebuilt in Coot to correct errors and extend unmodeled regions, followed by iterative refinement in Phenix (Supplementary Table S1)^30^. This workflow provides a general strategy for determining the structures of other T6SS effector proteins that are critical for *Francisella* replication and virulence (Fig. 1C).

### Domain organization and inter-domain interactions of PdpC

**Fig. 2:**
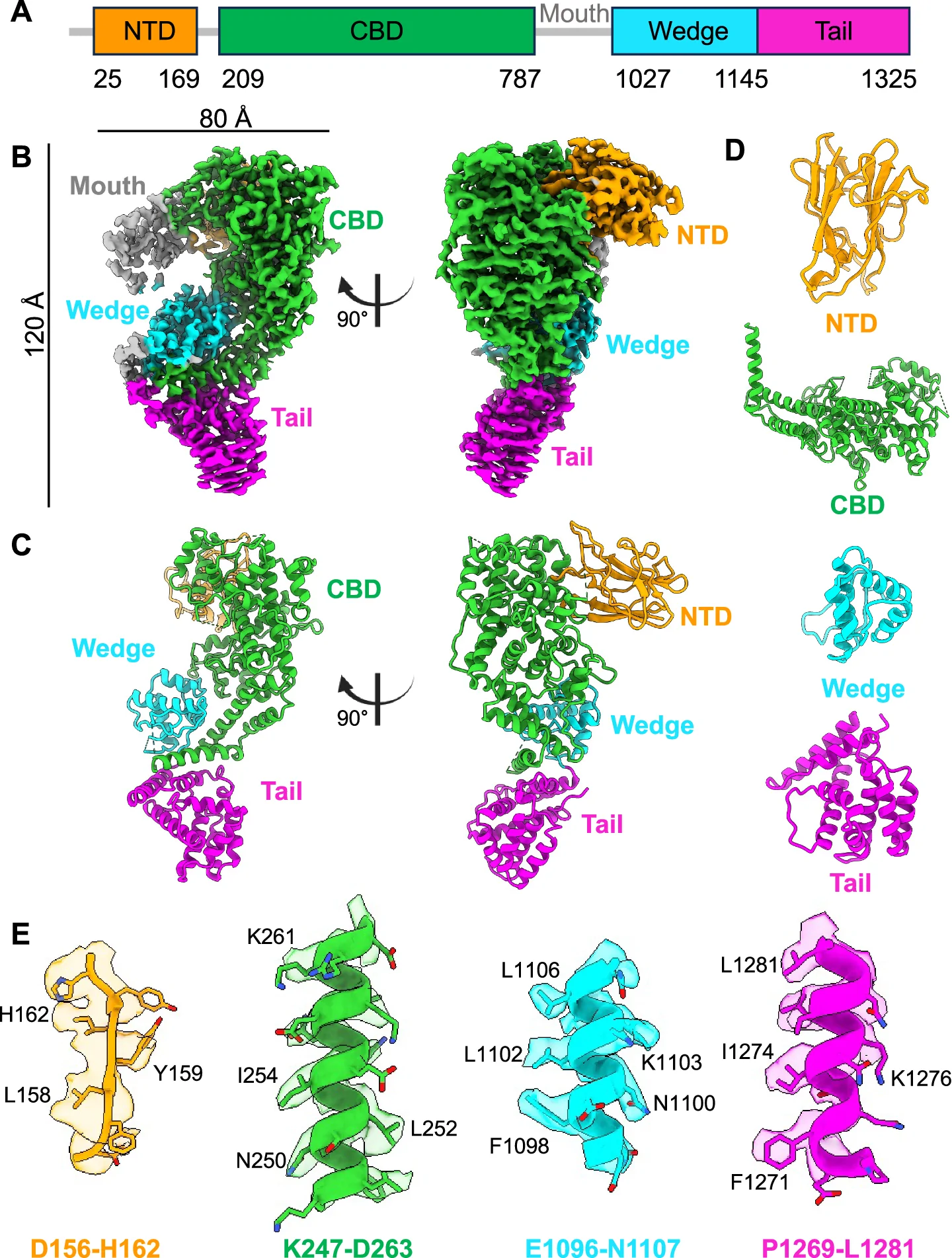
CryoEM structure of PdpC. **A** Domain organization of *Francisella* PdpC. Domains are labeled with different colors; unmodeled regions are shown in gray. **B** Two different views of the cryoEM density map of PdpC, with domains colored according to scheme in (**A**). **C** Two different views of the PdpC structure, colored as in (**B**). The N- and C-termini are indicated by black dots. **D** Ribbon representation of individual domains. **E** Representative density map.

The seahorse-shaped PdpC (Fig. 2) measures approximately 120 Å in height and 80 Å in width (Fig. 2A, B). The structure is primarily composed of α-helices, except for the N-terminal domain (NTD), which adopts a compact immunoglobulin (Ig)-like β-sandwich fold (Fig. 2C, D). PdpC can be divided into five sequential domains from the N-terminus to the C-terminus: the NTD (residues 25–169), which forms a prominent lateral head lobe; a central body domain (CBD, residues 209–787); an unmodeled region (residues 787–1027), referred to here as the “mouth” domain due to its location; a wedge domain (residues1027–1145), which locates at the groove of the body domain; and a C-terminal tail domain (residues 1145–1325), forming the tip of the molecule and completing the seahorse-like shape. While most of the structure was well resolved (Fig. 2E), a central ~200-residue region corresponding to the mouth domain lacked interpretable density, likely due to conformational flexibility, and could not be modeled. This segment is surrounded by the curved body, wedge, and tail domains, with the NTD extending laterally from the main structure. Importantly, structural homology searches revealed no similarity to any known protein in the PDB. These findings indicate that PdpC represents a novel protein fold of potential mechanistic and evolutionary significance.

Detailed examination of the individual domains highlights the structural uniqueness of PdpC (Fig. 2D). The NTD forms an Ig-like β-sandwich, composed of two β-sheets, and projects laterally from the main scaffold. The central body domain, the largest of the five, forms the structural scaffold of the protein, consisting entirely of α-helices arranged in a helical solenoid-like fold. It forms the “spine” of the seahorse-like shape, connecting all other domains and potentially contributing to the structural rigidity of the protein. The wedge domain, a compact α-helical module, nestles at the bottom of the body domain. The C-terminal tail domain is composed of several tightly packed α-helices forming a small, globular structure that caps the extended molecule. Two loops from the NTD insert into a groove on the CBD, with hydrophobic residues mediating their interactions (Supplementary Fig. S2A). The wedge domain forms extensive contacts with the CBD, with approximately half of the wedge domain enveloped by the CBD in a bay-like configuration (Supplementary Fig. S2B). The tail and CBD are brought together by two helices, one contributed by each domain, primarily stabilized by hydrophobic interactions (Supplementary Fig. S2C). Together, these features define PdpC as a modular, uniquely folded effector with no known structural homologs, reflecting evolutionary adaptation to its specialized role in *Francisella*’s T6SS.

### PdpC selectively binds phospholipids

**Fig. 3:**
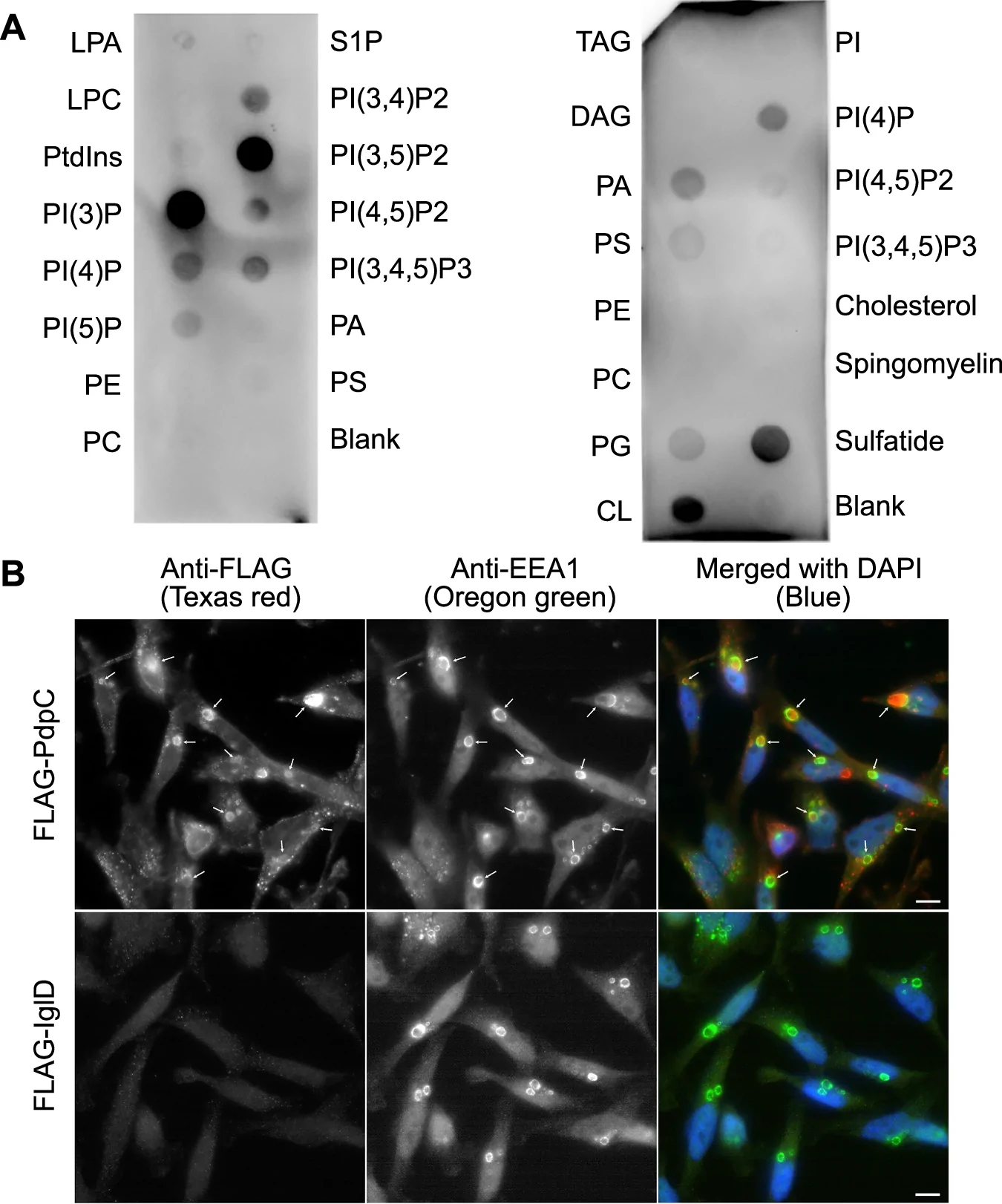
Phospholipid binding and subcellular binding distribution of PdpC. **A** Phospholipid binding of PdpC. FLAG-PdpC binds PI(3)P and PI(3,5)P2 as shown in the panel on the left and sulfatide and cardiolipin (CL) as shown on the right. LPA lysophosphatidic acid, LPC lysophosphatidylcholine, PtdIns, phosphatidylinositol, PE phosphatidylethanolamine, PC phosphatidylcholine, S1P, sphingosine-1-phosphate, PA phosphatidic acid, PS phosphatidylserine, TAG triacylglycerol, DAG diacylglycerol, PG phosphatidylglycerol, PI phosphatidylinositol. **B** Staining of HeLa-Rab5c Q79L cells by FLAG-PdpC. HeLa-Rab5c Q79L cells were fixed, permeabilized, and stained with either FLAG-PdpC (top panels) or, as a control, FLAG-IglD (bottom panels). Early endosomes were visualized by staining with mouse anti-EEA1 antibody. Nuclei were visualized by staining with DAPI. Arrows indicate colocalization of FLAG-PdpC and EEA1. Scale bars, 10 µm.

PdpC is essential for the intracellular replication and pathogenicity of *Francisella*, yet its mechanistic role remains unclear. Based on the knowledge that many bacterial toxins and secreted effector proteins interact with host lipids^31,32^, we examined whether PdpC binds specific lipids. Purified FLAG-tagged PdpC was incubated with phospholipids immobilized on nitrocellulose strips (Echelon). Intense chemiluminescent signals of FLAG-PdpC chemiluminescence were observed for PI(3)P, PI(3,5)P_2_, sulfatide and cardiolipin (Fig. 3A). Much weaker signals were observed for PI(4)P, PI(3,4)P_2_, PI(4,5)P_2_, and PI(3,4,5)P_3_ (Fig. 3A). Little to no signal was observed for phosphatidylinositol, cholesterol, phosphatidic acid, phosphatidylserine, phosphatidylethanolamine, phosphatidylcholine, triacylglycerol, diacylglycerol, sphingomyelin, and sphingosine-1-phosphate (Fig. 3A). These results demonstrate that PdpC binds selectively to a subset of phospholipids, including PI(3)P, a hallmark lipid of early endosomal and phagosomal membranes.

To determine whether PdpC binds biological membranes in cells, we examined the subcellular localization of PdpC after incubation of the FLAG-epitope tagged purified protein with formaldehyde-fixed, permeabilized eukaryotic cells. We observed that the staining pattern was subtle and suspected that head groups of signaling phospholipids are masked by binding to host cell proteins, such as FYVE and PX-domain containing proteins which would bind to and mask PI(3)P. To increase the abundance and availability of phospholipid head groups, we used HeLa cells producing GTPase-deficient Rab5c Q79L, a mutation that leads to persistent recruitment of PI3-kinases to early endosomes, generating large, distinctive early endosomes^33^. We chose this cell line because *Francisella* retards the maturation of its phagosome and its secreted effector OpiA has been shown to be a PI3-kinase^18^ that would generate PI(3)P on the *Francisella* phagosome. Immunofluorescence microscopy revealed that the FLAG-epitope tagged PdpC colocalized with large early endosomes (Fig. 3B, top panel). In contrast, no colocalization of FLAG-epitope staining was seen after incubating the HeLa Rab5c Q79L cells with the control epitope tagged protein, FLAG-IglD^34^ (Fig. 3B, bottom panel).

Whereas the *Francisella* T6SS secreted effector OpiA, has been shown to be a PI3-kinase^18^, we did not detect phosphoinositide kinase or phosphatase activity in PdpC under the conditions tested (Supplementary Fig. S3). Nonetheless, such activity might require specific cofactors or assay conditions other than those we tested.

### PdpC interacts with PdpE

After characterizing the lipid-binding properties of PdpC, we next investigated its potential protein partners among other FPI-encoded proteins. The *Francisella* genus is divided into three clades based on pan-genomic analysis^35^ with Clade A comprising species pathogenic to humans and terrestrial animals (including *tularensis* and *novicida*), and Clades B and C comprising environmental and water-related species (including *noatunensis*, *philomiragia*, *salina*, *uliginis*, and *frigidituris*). Notably, PdpC is encoded on the FPI of most strains of Clade A but is absent from all strains of Clades B and C. Interestingly, within the bacterial genome, *pdpE* is located immediately downstream of *pdpC*, and the two genes are consistently co-present or co-absent across all *Francisella* genomes analyzed.

**Fig. 4:**
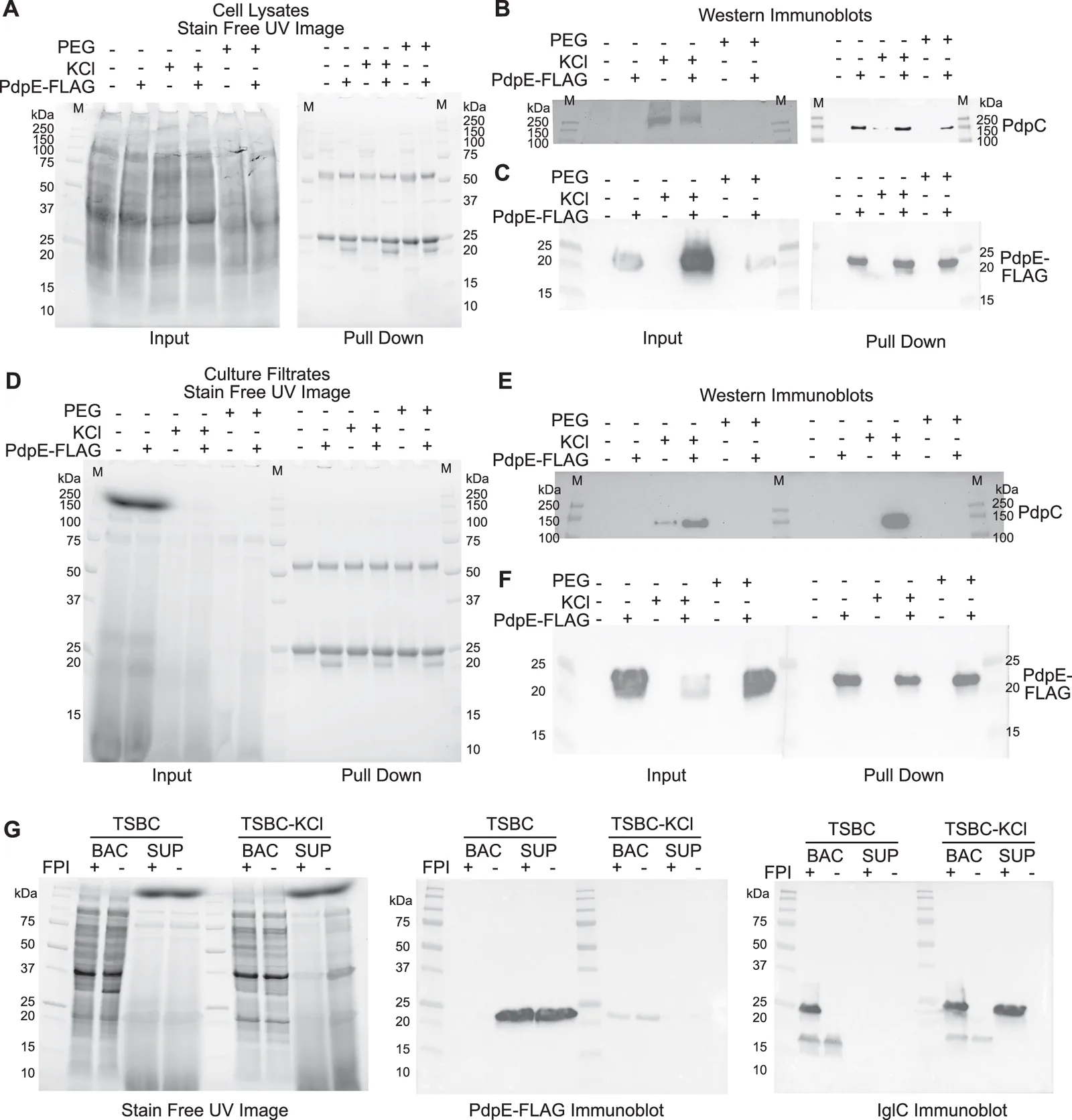
PdpE-FLAG pulls down PdpC in bacterial cell lysates and culture filtrates, and is secreted by a non-T6SS pathway. Fn wild-type (WT) strain and a strain producing PdpE-FLAG were grown either in TSBC, TSBC with 5% KCl, or TSBC with 10% PEG. Ultracentrifuge clarified bacterial cell lysates (**A**–**C**) and culture filtrates (**D**–**F**) were incubated with anti-FLAG agarose resin. Inputs and proteins pulled down by the anti-FLAG resin were analyzed by stain free UV image (**A**, **D**) and by Western Immunoblots for PdpC (**B**, **E**) and PdpE-FLAG (**C**, **F**). The 50 kDa and 25 kDa protein bands in (**D**) are immunoglobulin heavy and light chains. **G** PdpE-FLAG producing Fn strains with or without deletion of their FPI were cultured in TSBC or TSBC containing 5% KCl and ultracentrifuge clarified bacterial lysates (BAC) and concentrated culture supernates (SUP) were prepared for SDS-PAGE and Western blot analysis.

We examined the interaction between PdpC and PdpE using pulldown assays. PdpC-PdpE association was detected in clarified bacterial lysates (Fig. 4A–C) and culture filtrates (Fig. 4D–F). This observation is consistent with the report by Adams et al.^36^, who further proposed that PdpE acts as an effector protein that modulates and limits the toxicity of PdpC to infected host cells. Over-production of PdpE-FLAG in *F. novicida* led to a striking increase in PdpC levels in the culture filtrate (Fig. 4E, fourth vs. third protein input lanes), while cytosolic PdpC levels remained comparable (Fig. 4B, fourth vs. third input lanes). This may reflect stabilization of PdpC by PdpE in the culture filtrate – again consistent with a functional interaction between the two proteins.

To determine whether PdpE interaction with PdpC alters the lipid-binding capacity of PdpC, we purified PdpE-ALFA (Supplementary Fig. S4A) and PdpC-FLAG (Supplementary Fig. S4B) by affinity chromatography and incubated purified PdpC-FLAG with a 360-fold molar excess (50-fold mass excess) of purified PdpE-ALFA. No difference in PdpC-lipid binding was observed in the presence or absence of PdpE (Supplementary Fig. S4C).

Treatment with high KCl, which enhances T6SS-dependent expression and secretion, increased levels of PdpC (Fig. 4B, E) and IglC^10^ (Fig. 4G), but caused a dramatic decrease in secreted PdpE (Fig. 4F, G). Analysis of the PdpE sequence by Signal P6.0^37^ predicts the presence of a Type I signal sequence, suggesting secretion via the Sec-mediated pathway. Indeed, PdpE-FLAG was detected in the culture filtrate even in a *F. novicida* strain lacking the entire FPI^24^ (Fig. 4G). Thus, whereas PdpC secretion is stimulated under T6SS-inducing conditions, PdpE follows a distinct, FPI-independent secretion pathway.

### CBD of PdpC interacts with phospholipids and PdpE

To ascertain which domain of PdpC mediates phospholipid and PdpE binding, we expressed the coding sequence of the N-terminal domain (NTD) and central body domain (CBD) with ALFA-epitope tags. The NTD was highly prone to aggregation, preventing reliable biochemical analysis. In contrast, the CBD-ALFA construct was soluble and used for binding examination (Supplementary Figs. S5A–D, S6). We first examined its interaction with phospholipids in ELISA binding assays. Similar to full length PdpC (Fig. 3B), the CBD binds specifically to PI(3)P and to a lesser extent to cardiolipin (CL) and sulfatide, but not to phosphatidylcholine (PC) or phosphatidyl serine (PS) (Supplementary Fig. S5A). We examined whether this binding might result from non-specific electrostatic interactions. As both CBD and CBD-ALFA have a slightly acidic iso-electric point (pI) of 6.4, electrostatic interactions would be expected with positively charged phospholipid headgroups such as lecithin rather than the negatively charged PI(3)P, CL, and sulfatide. However, binding to PI(3)P, CL, and sulfatide was unaffected by the presence of 0.45 M NaCl (Supplementary Fig. S5A), indicating that the interaction is specific and not electrostatically driven. We next tested whether the CBD also interacts with PdpE. We performed a pulldown assay using *F. novicida* producing both PdpC CBD-ALFA and PdpE-FLAG (Supplementary Fig. S5B–D). Similarly, just as we observed that full length PdpC interacts with PdpE, we also observed that ALFA-epitope tagged CBD pulled down PdpE-FLAG (Supplementary Fig. S5B–D). Thus, the CBD is sufficient to mediate both phospholipid and PdpE binding, although other regions of PdpC may also contribute.

PdpE-FLAG runs as a doublet on SDS-PAGE (Supplementary Fig. S5D), with a major band at 23.6 kDa in the input lanes consistent with the size of the protein after Sec signal peptide cleavage (22.1 kDa) and correction for its acidic pI (4.9) using the equation of Guan et al.^38^. The higher molecular weight band at 26 kDa, which is fainter in the input lane but stronger in the CBD pulldown lane (red arrowhead, Supplementary Fig. S5D), is consistent with the unprocessed, full length PdpE-FLAG (24.4 kDa) corrected for its acidic pI using the Guan et al. equation^38^. That the CBD primarily pulls down the higher molecular weight, unprocessed PdpE is consistent with the CBD-PdpE interaction occurring predominantly in the bacterial cytosol prior to secretion. In contrast, pull down assays with full length PdpC recovered mainly the processed form of PdpE (Fig. 4C, F), indicating an interaction occurring after translocation of PdpE into the periplasm or extracellular milieu.

## Discussion

The primary sequence and atomic structure of many bacterial effectors, including *Francisella* PdpC, frequently fail to predict their pathophysiological functions^39^, underscoring the complexity of effector-mediated disease mechanisms, particularly in tularemia. Indeed, the molecular mechanisms by which *F. tularensis* escapes the phagosome and establishes a replicative niche in the host cytosol have remained enigmatic despite the identification of the FPI and its T6SS more than 15 years ago^7,8^. PdpC, one of the few bona fide T6SS effectors whose deletion impairs phagosomal escape and virulence^13–17^, has been a particularly elusive target. In this study, we determine the first atomic view of PdpC and uncover two interacting features that explain its spatiotemporal action during infection.

The 3.4 Å cryoEM structure reveals a highly elongated, seahorse-shaped monomer with a completely novel fold—no structural homologs were identified by DALI, and AlphaFold predictions failed to anticipate the observed architecture. The large central body domain (CBD) forms an extended α-helical scaffold that integrates an N-terminal Ig-like domain, a flexible “mouth” region, a wedge domain, and a C-terminal tail. Strikingly, the CBD alone is sufficient for the two key biochemical activities we identified: selective binding to host phospholipids—most prominently PI(3)P—and direct interaction with PdpE.

**Fig. 5:**
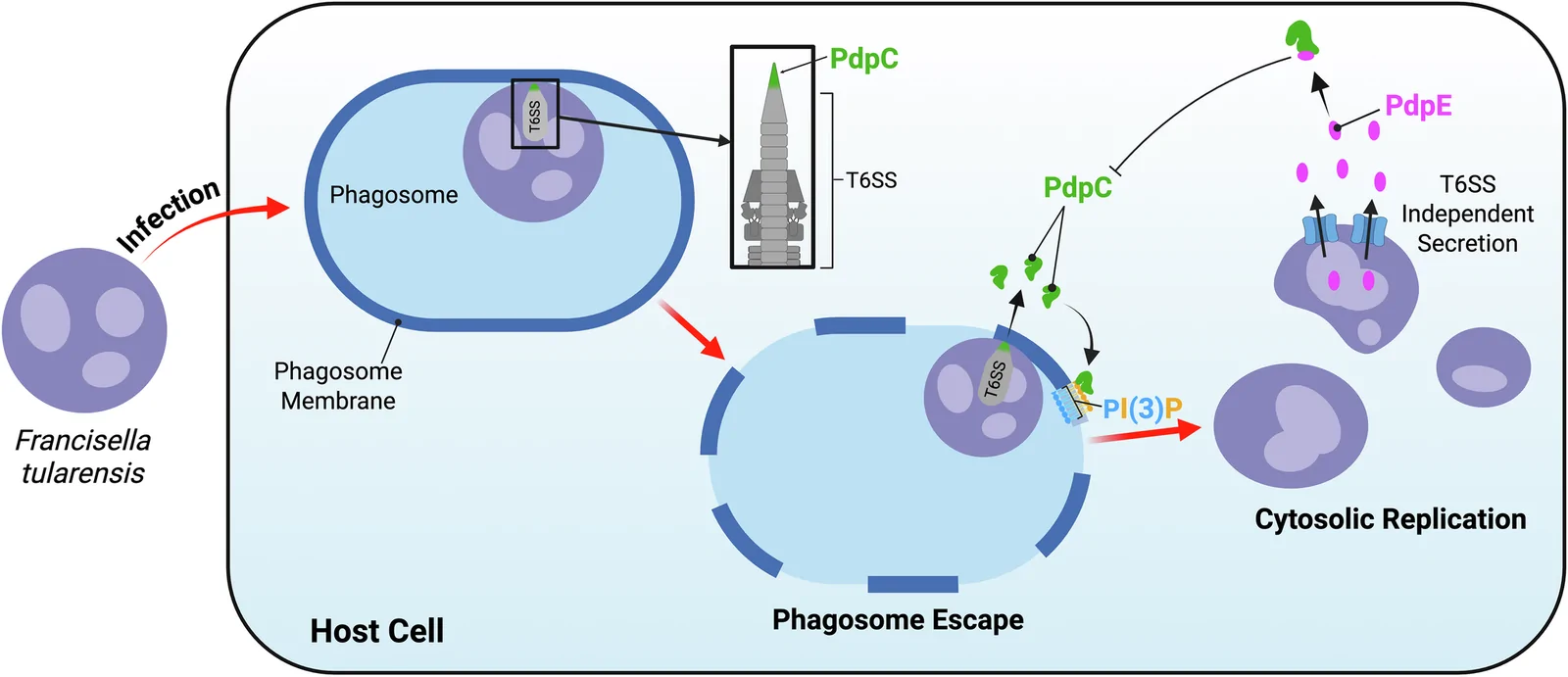
Proposed model of PdpC binding to PI3P and PdpE in host cells. Key stages of *Francisella* lifecycle within a host cell is illustrated, highlighting components analyzed in this study, including: T6SS, PdpC, PI(3)P and PdpE. Red arrows indicate the sequential progression beginning with infection of the host cell by uptake into a phagosome. PdpC is secreted through the bacterium’s T6SS and binds to the specific host cell phospholipid PI(3)P on the early phagosomal membrane. This binding activity anchors the effector protein on the cytosolic side of the *Francisella* phagosome, exerting its activity to promote phagosome escape. *Francisella* replicates within the host cytoplasm. PdpE, a protein secreted via a T6SS-independent pathway, directly interacts with PdpC. This interaction may reduce PdpC-induced cellular toxicity, thereby facilitating host cell bacterial replication. Note: Depictions are not drawn to scale. The figure was created in BioRender https://BioRender.com/rh2r587.

PI(3)P is a signature lipid of the cytosolic leaflet of early phagosomes. Our lipid binding assay demonstrated that PdpC selectively binds to PI(3)P, PI(3,5)P_2_, sulfatide and cardiolipin, but not to PI(4)P, PI(3,4)P_2_, PI(4,5)P_2_, and PI(3,4,5)P_3_. Consistent with our findings, Horzempa’s lab also reported PdpC binding to PI(3)P, as documented by Schrieber^40^. Since PI(3)P is localized to the cytosolic face of early phagosomes, the selective binding of PdpC to PI(3)P may serve to anchor PdpC to the *Francisella* phagosome, thereby exerting its (still unknown) enzymatic or disruptive actions locally rather than diffusely throughout the cell (Fig. 5). It is noteworthy that phosphoinositide binding is common among bacterial toxins and effector proteins with diverse biological functions^31,41–44^. Targeted delivery by binding to phosphoinositides is employed by multiple *Legionella* T4SS effectors to achieve precise membrane engagement^45^. The *Legionella* secreted effector proteins RavD^46^ and LtpM^47^ have been shown to bind PI(3)P but their sequences and structures (PDB 6NJD and 8XGQ, respectively) have no similarity to the sequence or structure of PdpC. Beyond bacterial secreted effectors and toxins, oomycete plant pathogens export effector proteins (such as Avr1b) that enter plant cells by binding to PI(3)P on the surface of the cells via N-terminal RxLR and dEER motifs^48^. While PdpC has an RSLR sequence (residues 57–60), it lacks a dEER motif and does not share significant sequence homology with Avr1b.

The additional binding of PdpC to PI(3,5)P₂, sulfatide, and cardiolipin suggests it may sense or influence later endolysosomal compartments or stress-induced mitochondrial membranes, potentially broadening its role in delaying phagosome maturation or modulating autophagy^49–51^.

A second layer of regulation is provided by PdpE, an FPI-encoded protein secreted via a T6SS-independent (likely Sec-mediated) pathway. We show that PdpE physically interacts with the same CBD of PdpC that binds PI(3)P. Remarkably, the two proteins are secreted by distinct routes with opposing regulation: T6SS-dependent PdpC release is strongly induced by high KCl, whereas PdpE secretion is repressed under the same conditions. This reciprocal control creates a temporal switch (Fig. 5): during the early phagosomal stage, unopposed PdpC is delivered by the T6SS and tethered to PI(3)P-rich membranes to drive escape; after cytosolic replication begins, PdpE accumulates extracellularly and binds PdpC, likely attenuating its toxicity and enabling additional rounds of replication as recently proposed^36^. The physical separation of secretion pathways prevents premature PdpC–PdpE complex formation inside the bacterium, ensuring that antagonism occurs only after escape. Premature interaction of PdpC with PdpE in the bacterial cytosol may be prevented by partitioning chaperones/secretion pilots, as in the case of *Salmonella enterica* T3SS effector proteins SipB and SipC, whose premature association is prevented by the chaperonin/secretion pilot SicA^52^. Alternatively, PdpE could be co-translationally secreted by the Sec pathway, thereby preventing interaction between PdpE and PdpC in the bacterial cytosol. Thus, PdpC and PdpE constitute a sophisticated effector duo: one partner (PdpC) is fired by the T6SS to breach the phagosome, while the second (PdpE) is released independently to rein in PdpC effector activity once the job is done. This coordinated, two-component strategy represents a new paradigm among T6SS effectors.

While standard bio-informatic tools do not demonstrate any convincing homology with proteins outside of *Francisella*, Broms et al.^25^ recently reported that a region of 335 amino acids of PdpC (residues 445–779) share sequence homology with a 319 residue region of a hypothetical *Legionella gratiana* protein (WP_058499943.1). The latter is a 1741 residue protein predicted to have protein kinase activity, but the region of sequence similarity lies outside of the predicted kinase region of the hypothetical *L. gratiana* protein. Jackhmmer search identified more distant homologs and a conserved region found in Makes Caterpillars Floppy (MCF) domain containing bacterial toxins. Highly conserved residues within this region of homology include a serine, histidine, and glutamate triad (termed a “MCF-SHE” consensus sequence). Alanine substitutions of these conserved residues in the *F. tularensis* Live Vaccine Strain (LVS) did not alter PdpC secretion but resulted in marked decreases in cytotoxicity (decreased LDH release) and intracellular replication in macrophages and loss of virulence in a mouse model of infection. These authors suggested the conserved SHE residues as a catalytic triad and our structure confirms an alignment of side chains consistent with such a function (Supplementary Fig. S7). Further studies are needed to determine the validity and nature of the proposed catalytic activity.

The unique seahorse fold, the exposed lipid-binding CBD, and the discrete PdpE interaction surface now provide concrete footholds for mechanistic dissection. Structure-guided mutants can test whether PI(3)P binding is required for phagosomal targeting, whether the flexible mouth domain harbors catalytic activity, and whether PdpE binding occludes the toxic moiety of PdpC. Moreover, the complete absence of structural homologs outside *Francisella*, combined with its essential role in a Tier 1 select agent, makes PdpC an attractive target for therapeutics that could neutralize tularemia at its critical escape step.

One limitation of our studies is that they have been done using epitope tagged proteins, which could alter the properties of the proteins. Nonetheless, in the case of lipid binding properties of PdpC, our results are similar to those reported by Schrieber^40^ for the native protein using a rabbit anti-PdpC polyclonal antibody. The combination of our results with those of Schrieber^40^ strengthens the conclusions, since the latter used bacterial lysates and a polyclonal antibody which can cross react with other proteins, whereas our study used purified epitope tagged PdpC and a specific monoclonal antibody against the epitope tag. With regard to PdpE, we have demonstrated that PdpE-FLAG pulls down native, untagged PdpC and that CBD-ALFA pulls down PdpE-FLAG. It would be unlikely for these orthogonally congruent results to be an artefact of epitope tagging.

By solving the first structure of a *Francisella* T6SS effector and revealing a temporally regulated effector–anti-effector pair, this work transforms PdpC from an orphan virulence factor into a tractable molecular switch at the heart of *Francisella*’s intracellular lifestyle, opening new avenues for both fundamental cell biology and anti-infective development against one of the most virulent bacterial pathogens known.

## Methods

### Plasmid construction

For construction a vector producing His_6_-FLAG-PdpC, the promoter sequence of bacterioferritin (Pbfr) was amplified by PCR using genomic DNA prepared from *F. tularensis* subsp. *holarctica* Live Vaccine Strain. DNA sequences for a histidine tag (HHHHHH) followed by a thrombin cleavage site (LVPRGS) and a FLAG tag (DYKDHDGDYKDHDIDYKDDDDK) followed by a TEV protease cleavage site (ENLYFQG) were added to the 3’-end of Pbfr sequence by overlap extension PCR to generate the Pbfr-His_6_-FLAG fragment. The sequence corresponding to nucleotides 4–3978 of pdpC (FTN_1319, 1325 amino acids) was amplified using *F. novicida* U112 genomic DNA as template. The rrnBT1/T2 terminator sequence was amplified from pDONR207 SARS-CoV NSP1, a gift from Fritz Roth^53^ (Addgene Plasmid #141255). These three PCR fragments were treated with appropriate restriction enzymes and cloned into pMP633 vector^54^.

For the domain-based lipid binding study, an expression cassette containing the Pbfr and nucleotide sequence corresponding to PdpC CBD (residues 209–787) with an ALFA tag (SRLEEELRRRLTE) at its C-terminus was cloned into pMP633. For the domain-based pulldown study, an expression cassette containing the Pbfr and nucleotide sequence corresponding to PdpC CBD with or without an ALFA tag at its C-terminus along with a second expression cassette containing the promoter of a major *F. novicida* outer membrane protein (FTN_1451) and the nucleotide sequence coding PdpE with a C-terminal FLAG tag were cloned into the pMP633 vector. *F. novicida* strain expressing the coding sequence of PdpE with a C-terminal FLAG tag or an ALFA tag driven by the Pbfr in pMP633 was used in pulldown and lipid binding studies of the full-length PdpC. Plasmid constructs were verified by whole plasmid sequencing (Plasmidsaurus).

### Production and purification of PdpC

*F. novicida* strain U112 producing N-terminal His_6_-FLAG epitope tagged PdpC was inoculated into 3 liters of Trypticase Soy Broth with 0.2% cysteine (TSBC), 5 mM betaine, 0.1 mg/mL FeSO_4_, 0.2 mg/ml hygromycin, containing 5% KCl to induce formation of the T6SS^10^. The bacteria were grown at 37 °C with shaking at 140 rpm to an optical density (540 nm) of 2, pelleted by centrifugation, resuspended in 100 mL of lysis buffer [20 mM HEPES, pH 7.5, 0.3 M NaCl, 1 mM EDTA, 3 mM MgCl_2_, 10 mM Imidazole, 0.8% CHAPS, and 10% glycerol, 1 mM PMSF, 1 mM N-ethylmaleimide, and 1:100 dilution of Protease Inhibitor Cocktail (MedChem Express, HY-K0010)]. The bacteria were lysed by sonication with a probe tip sonicator (Ultrasonics W-375) while cooling on ice and the sonicate was clarified by ultracentrifugation at 44,400 × *g* for 90 min. The clarified supernatant fluid was added to 1 mL of Ni-agarose (Pierce High-Capacity Ni-IMAC Resin), rotated end-over-end overnight at 4 °C, washed with lysis buffer, transferred to a column, and eluted with lysis buffer containing 20 mM, 50 mM, 100 mM, and 250 mM imidazole (10 mL each) with collection of 3 mL fractions. The eluted fractions were analyzed by SDS-PAGE and fractions containing PdpC were combined, concentrated with a 100,000 MWCO centrifugal filter concentrator (Amicon Ultra-15) and applied to a Sephacryl S200 gel filtration column that had been pre-equilibrated with 20 mM HEPES, pH 7.5, 1 mM EDTA, 0.3 M NaCl, 10% glycerol, 1% CHAPS. Fractions were collected and analyzed by SDS-PAGE with protein staining by Sypro Ruby. Fractions with strong band at 150 kDa were pooled, concentrated with a 100,000 MWCO centrifugal filter concentrator and changed to a buffer containing 20 mM HEPES, 0.3 M NaCl, 3% glycerol, 0.1% CHAPS using a 40 kDa MWCO Zeba Spin Desalting column (Thermo Fisher) for use in cryoEM analysis.

### CryoEM sample preparation and image collection

For cryoEM sample preparation, we applied 3 μL of purified protein sample to the graphene oxide (GO)-coated EM grids (Quantifoil Au 1.2/1.3, 300 mesh) obtained from Shuimu BioSciences. The grid was incubated for 10 s and blotted for 12 s with filter paper (Ted Pella) at force 10, and then plunge frozen in liquid ethane using an FEI Vitrobot Mark IV (Thermo Fisher Scientific) set to 8 °C temperature and 100% humidity. CryoEM grids were loaded into a Titan Krios electron microscope (Thermo Fisher Scientific) operated at 300 kV, equipped with a Gatan K3 Summit direct electron detector and a GIF Quantum LS energy filter set to a slit width of 20 eV. Automated data collection was performed using SerialEM^55^. The total dose rate on the sample was set to ~50 e^−^/Å^2^/movie at a pixel size of 1.1 Å. In total, 39,482 movies were collected.

### Single-particle cryoEM reconstruction and atomic model building

Motion correction and defocus estimation were performed in Live CryoSPARC^56^, followed by subsequent data processing using cryoSPARC. Particles were picked with Topaz^57^, and then subjected to extensive 2D and 3D classification. 2,801,637 particles were selected and imported into Relion for further classifications. First round of 3D classification was performed with parameters K = 6 T = 4, 1,325,626 particles were selected. After second round of 3D classification without alignment, 616,877 particles retained. After ctf refine and 3D refinement, heterogeneous refinement was performed in CryoSPARC to remove low-quality particles, resulting in 322,627 particles retained for further data processing. To overcome the density distortion caused by preferred orientation, spIsoNet^28^ and the modified Blush regularization algorithm in RELION 5^58^ was applied to improve particle alignment accuracy. This optimized workflow ultimately produced a cryoEM map of PdpC at ~3.4 Å resolution with well-resolved side-chain features in representative regions that enable confident model building (Fig. 2E).

We first used ModelAngelo^29^ to generate an initial model. The atomic model of PdpC was then manually built and refined in COOT^59^, followed by further refinement in Phenix^30^. Refinement statistics of the model are summarized in Table 1. Figures were generated using USCF ChimeraX^60^.

**Table 1:** CryoEM data collection, refinement and validation statistics

	PdpC EMD-73168 PDB 9YNB
Data collection and processing	
Magnification	81,000
Voltage (kV)	300
Electron exposure (e^−^/Å^2^)	50
Defocus range (μm)	−1.8 to −2.6
Pixel size (Å)	1.1
Symmetry imposed	C1
Particle number	322,627
Map resolution	3.4
FSC threshold	0.143
Refinement	
Map sharpening *B* factor (Å^2^)	−116
Model composition	
Non-hydrogen atoms	7462
Protein residues	905
Ligand	
*B* factors (Å^2^)	
Protein	13.6
Ligand	
R.m.s. deviations	
Bond lengths (Å)	0.003
Bond angle (°)	0.690
Validation	
MolProbity score	1.72
Clashscore	7.10
Poor rotamers (%)	0.24
Ramachandran plot	
Favored (%)	95.29
Allowed (%)	4.71
Disallowed (%)	0

### Nitrocellulose strip lipid binding assay

Nitrocellulose strips with phospholipids (Phosphoinositide Interaction Protein (PIP) strips, Echelon) were blocked by incubation with Tris buffered saline with 1% BSA and 0.1% Tween-20 and incubated overnight at 4 °C with His_6_-FLAG-PdpC (5 μg/mL) in the blocking buffer. Strips were washed three times in TBS containing 0.1% Tween-20 and incubated with mouse monoclonal anti-FLAG-HRP (1:30,000, Thermo Fisher Scientific). Chemiluminescent signals were developed by incubating with Clarity Western ECL substrates (Bio-Rad) and detected using a ChemiDoc Imaging System.

### Phospholipid ELISA binding assay

*F. novicida* producing CBD-ALFA was grown overnight in TSBC containing 0.2 mg/mL hygromycin, pelleted by centrifugation, and resuspended in 1/14th of the original culture volume in TBS containing 0.5% TX-100, 1 mM EDTA, 1 mM NEM, 1 mM PMSF, and 1:100 protease inhibitor cocktail (MedChem Express), sonicated on ice, clarified by ultracentrifugation at 36,640 × *g* for 3 h at 4 °C, and diluted 50-fold with 20 mM Tris-HCl, pH 7.4, containing 0.1% Tween-20 and 1% BSA and either 0.15 M NaCl or 0.45 M NaCl for use in phospholipid binding assays. Phospholipid samples used for coating ELISA plate wells were prepared as follows: PI(3)P (Cayman Chemical) was dissolved in 20:9:1 chloroform:methanol:water (0.3 mg/mL); phosphatidylserine (Carbosynth LLC) was dissolved in chloroform (6.5 mg/mL); phosphatidylcholine (lecithin, Avanti Polar Lipids) was dissolved in chloroform:methanol:water (65:25:4) at 5 mg/mL; sulfatide (cerebroside sulfate, VWR) was dissolved in 9:1 chloroform:methanol at (1 mg/mL), and cardiolipin (Avanti Polar Lipids) was dissolved in chloroform:methanol:water (73:23:3) at 5 mg/mL. All lipids were then further diluted in 1:9 CHCl_3_:methanol to a final concentration of 17 µg/mL and 0.1 mL of this (or 0.1 mL of the 1:9 CHCl_3_:methanol vehicle control) was added to wells of a 96-well polystyrene ELISA plate (Costar cat #3370). The wells were allowed to evaporate to dryness in a fume hood, blocked with 1% BSA in TBS, washed with TBS containing, 1% BSA in TBS with 0.1% Tween-20, and incubated for 90 min at room temperature with CBD-ALFA prepared as described above. Wells were washed three times with TBS containing, 1% BSA in TBS with 0.1% Tween-20, incubated with HRP-conjugated alpaca single domain anti-ALFA antibody (1:4000, Synaptic Systems), washed with TBS containing 0.1% Tween-20, developed with TMB substrate (Thermo-Fisher Scientific), and the reaction stopped by addition of H_2_SO_4_ per manufacturer’s directions. Absorbance at 450 nm was measured with an iMark microplate reader (BioRad).

For ELISA based assays of full-length PdpC-FLAG binding to lipids, the ELISA plates were prepared, blocked and washed as described above, and incubated with PdpC-FLAG at a final concentration of 1 µg/mL for 90 min at room temperature in TBS containing 1% BSA and 0.1% Tween-20, washed, incubated for 90 min with HRP-conjugated mouse anti-FLAG monoclonal antibody M2 (1:10,000, Thermo Fisher); subsequent processing steps were performed as described above.

### PdpC-FLAG binding to subcellular compartments in HeLa-Rab5c Q79L cells

HeLa Tet-off cells (Clontech) stably transfected with tetracycline-regulated synthesis of GTPase deficient Rab5c Q79L cells were prepared and cultured as we have described previously^33^. The cells were plated onto poly-D-lysine coated glass coverslips in 24-well plates at a density of 1 ×10^5^ cells/well in Dulbecco’s Modified Eagles Medium (DMEM) with 10% heat inactivated fetal bovine serum. To optimize synthesis of the mutant Rab5c in the Tet-off cells, we used fetal bovine serum certified to be tetracycline free. After 48 h in tetracycline-free culture medium, the cells were fixed for 30 min in 4% paraformaldehyde in phosphate buffered saline (PBS), washed with PBS, and permeabilized for 30 min with 0.1% saponin in PBS containing 10 mM lysine. The coverslips were incubated for 90 min at room temperature with either 1 µg/mL FLAG-epitope-tagged-PdpC or, as a control, 1 µg/mL FLAG-epitope-tagged-IglD (prepared as described previously^34^) in 0.1% saponin and 0.1% bovine serum albumin in PBS; secondary staining used rabbit-anti-FLAG antibody (10 µg/mL, Invitrogen) and Texas Red-conjugated goat-anti-rabbit IgG (10 µg/mL, Thermo Fisher). Early endosomes were visualized by staining with mouse anti-EEA1 IgG (5 µg/mL, Abcam) and Oregon Green-conjugated goat anti-mouse IgG (10 µg/mL, Thermo Fisher). Nuclei were visualized by staining with 1 µg/mL DAPI (Thermo Fisher) in PBS. The monolayers were washed in PBS, mounted in Prolong Glass Antifade Mounting Medium (Thermo Fisher), and epifluorescence was examined using an Eclipse TE2000-S microscope equipped with an X-Cite 120 light source (Nikon) and Semrock Brightline DAPI, FITC, and Texas Red filter cubes. Images were acquired with a PCO Edge 26 sCMOS monochrome camera (Excelitas) operated by Micromanager-2.0 using pco.camware. The PdpC and IglD-control images were acquired using identical exposure and camera settings. Images were assembled with Adobe Photoshop software.

### PdpE-FLAG pulldown of PdpC

Wild type *F. novicida* and *F. novicida* producing PdpE-FLAG were grown overnight either in TSBC, TSBC with 5% KCl, or TSBC with 10% PEG 3350 (Sigma Aldrich). Bacteria were pelleted by centrifugation, resuspended in 1/14th the original culture volume in PBS containing 0.5% TX-100, 1 mM EDTA, 1 mM PMSF, 1 mM NEM, and 1:100 dilution of a protease inhibitor cocktail (MedChemExpress, HY-K0010), lysed by sonication, and clarified by ultracentrifugation (36,640 × *g*) for 3 h at 4 °C. The culture supernates from the overnight cultures were filtered through a 0.2 micron filter and equilibrated with PBS containing 1 mM EDTA by overnight dialysis (Spectrapor dialysis tubing, MWCO 10 kDa) at 4 °C. M2-Anti-FLAG agarose resin was added to the clarified culture lysates (10 µL resin per 1.4 mL of clarified lysate) and to the dialyzed culture filtrates (10 µL resin per 10 mL of culture filtrate) and the resin containing samples were rotated overnight at 4 °C and washed with PBS containing 0.5% TX-100 to remove non-specifically bound proteins prior to analysis by SDS-PAGE and Western Immunoblotting. For SDS-PAGE and Western immunoblot analysis of input samples, the proteins in the dialyzed TSBC and TSBC with 5% KCl culture filtrates were concentrated 50-fold with a 10 kDa MWCO filter (Amicon Ultra-15). For SDS-PAGE analysis of the TSBC with 10% PEG culture filtrate input sample (which cannot be concentrated by a spin filter concentrator), the proteins were precipitated by addition of 100% trichloroacetic acid (TCA) containing 4 mg/mL deoxycholate to achieve a final concentration of 25% TCA, incubating on ice for 30 min, centrifugation at 10,000 × *g* for 30 min, washing the pellet in −20 °C acetone to remove residual TCA, and resuspending the precipitated proteins in a volume of SDS-PAGE sample buffer corresponding to 1/50th the original culture filtrate volume. PdpE-FLAG was detected using HRP-conjugated M2 anti-FLAG antibody (1:30,000, Sigma Aldrich) and PdpC was detected using polyclonal rabbit anti-PdpC antibody (1:1000, BEI Resources) and HRP conjugated goat anti-rabbit IgG antibody (1:30,000, BioRad) and chemiluminescent substrate (BioRad).

### CBD-ALFA pulldown of PdpE-FLAG

*F. novicida* producing PdpE-FLAG or both PdpE-FLAG and CBD-ALFA were grown overnight in TSBC with hygromycin 200 μg/mL. The bacteria were pelleted and ultracentrifuge clarified lysates prepared as described above. The clarified lysates (1.4 mL) were incubated with 10 µL of anti-ALFA agarose resin overnight to pulldown CBD-ALFA and its interacting proteins. The resin was washed extensively with TBS containing 0.1% TX-100 and the input and pulldown samples were analyzed by SDS-PAGE and Western immunoblotting using HRP-conjugated antibodies directed against the ALFA and FLAG epitope tags.

### Phosphoinositide kinase and phosphatase assay

Epitope tagged PdpC (0.2 mg/mL), purified as described above, was incubated with 50 µM BODIPY-fluorescent labeled PI, PI(3)P, or PI(3,5)P_2_ PIPES (Echelon Biosciences) in 0.1 M PIPES, pH 7.0, 2 mM MgCl_2_, 0.5 mM MnCl_2_, with or without 10 mM ATP, 10 mM MgCl_2_, for 90 minutes at 37 °C in total volumes of 25 µL. Reactions were stopped by the addition of 5 volumes of acetone, and the samples were dried under vacuum. BODIPY-labeled lipids were extracted with CHCl_3_, spotted onto silica thin layer chromatography plates, developed in CHCl_3_:methanol:acetone:acetic acid:water (70:50:20:20:20), and imaged under blue light with a BioRad ChemiDoc Imaging System.

### Statistics and reproducibility

Western Immunoblots, nitrocellulose strip lipid binding assays, immunofluorescence, and thin layer chromatography experiments were done twice with similar results. Phospholipid ELISA binding assays were done twice in quadruplicate and statistical comparisons were performed by two-way ANOVA using GraphPad Prism v. 9.2 with Šídák’s correction for multiple comparisons.

### Reporting summary

Further information on research design is available in the Nature Portfolio Reporting Summary linked to this article.

## Supplementary information

Supplementary information

Reporting Summary

## Data Availability

The cryoEM density map has been deposited in the Electron Microscopy Data Bank under accession code 9YNB. The atomic coordinate has been deposited in the Protein Data Bank under accession code EMD-73168. Uncropped gels and blots, as well as the source data for Supplementary Figs. S4C and S5A, are provided in the Supplementary Information as Supplementary Tables 1 and 2.
