# Supplementary material for: Structure and potential role of T6SS effector PdpC in Francisella tularensis intracellular lifestyle
Source: Commun Biol. 2026 Jun 25;9:1352. doi: 10.1038/s42003-026-10543-y (PMC13669386; doi:10.1038/s42003-026-10543-y)
Supplement: Supplementary file 1 [file 42003_2026_10543_MOESM1_ESM.pdf]

Structure and potential role of T6SS effector PdpC in *Francisella tularensis* intracellular lifestyle

Xiaoyu Liu[1,2,3,*], Daniel L. Clemens[4,*], Bai-Yu Lee[4,*], Xian Xia[2,3,5], Hongcheng Fan[2,3], Kaelyn Y. Feng[2,3], Marcus A. Horwitz[2,4,#], and Z. Hong Zhou[2,3,#]

[1]School of Life Sciences, Central China Normal University, Wuhan, 430079, China

[2]Department of Microbiology, Immunology and Molecular Genetics, University of California, Los Angeles (UCLA), Los Angeles, CA 90095, USA;

[3]The California NanoSystems Institute (CNSI), UCLA, Los Angeles, CA 90095, USA;

[4]Department of Medicine, University of California, Los Angeles (UCLA), Los Angeles, CA 90095, USA

[5]State Key Laboratory of Virology and Biosafety, College of Life Sciences, TaiKang Center for Life and Medical Sciences, Wuhan University, Wuhan 430072, Hubei, China

* These authors contributed equally.

[#]Corresponding authors: Hong.Zhou@ucla.edu (cryoEM and structures) and MHorwitz@mednet.ucla.edu (infectious diseases and biochemistry).

SUPPLEMENTARY FIGURES:

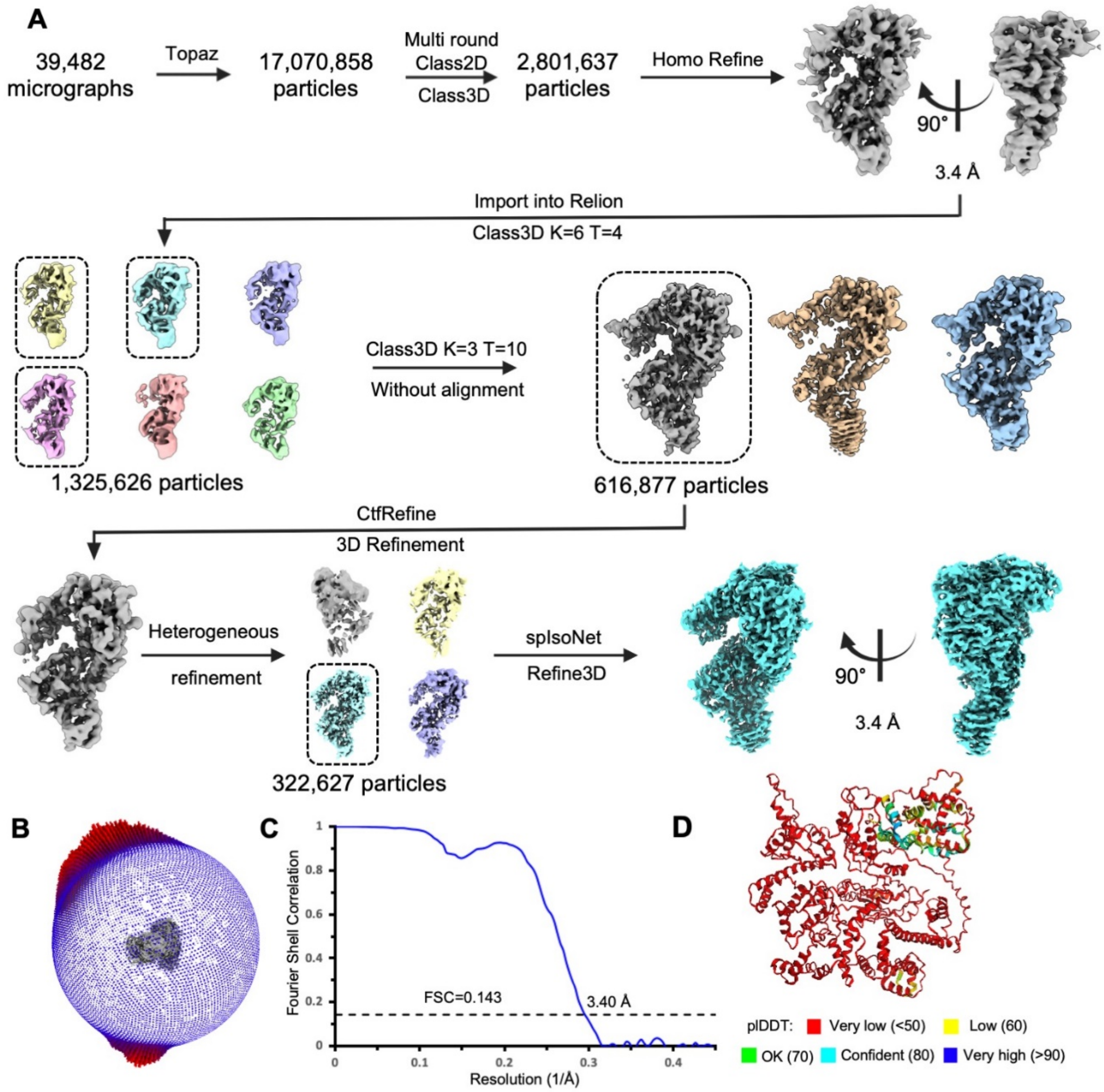

Supplementary Fig. S1: Data processing and AlphaFold prediction of PdpC*. (*A) Flowchart of cryoEM data processing. **(B)** Angular distribution of particles used in the final reconstruction. **(C)** Gold-standard Fourier shell correlation (FSC) curves for cryoEM map of PdpC. **(D)** AlphaFold prediction of PdpC, colored by pLDDT. The prediction shows low confidence and poor correlation with the cryoEM structure determined in this study.

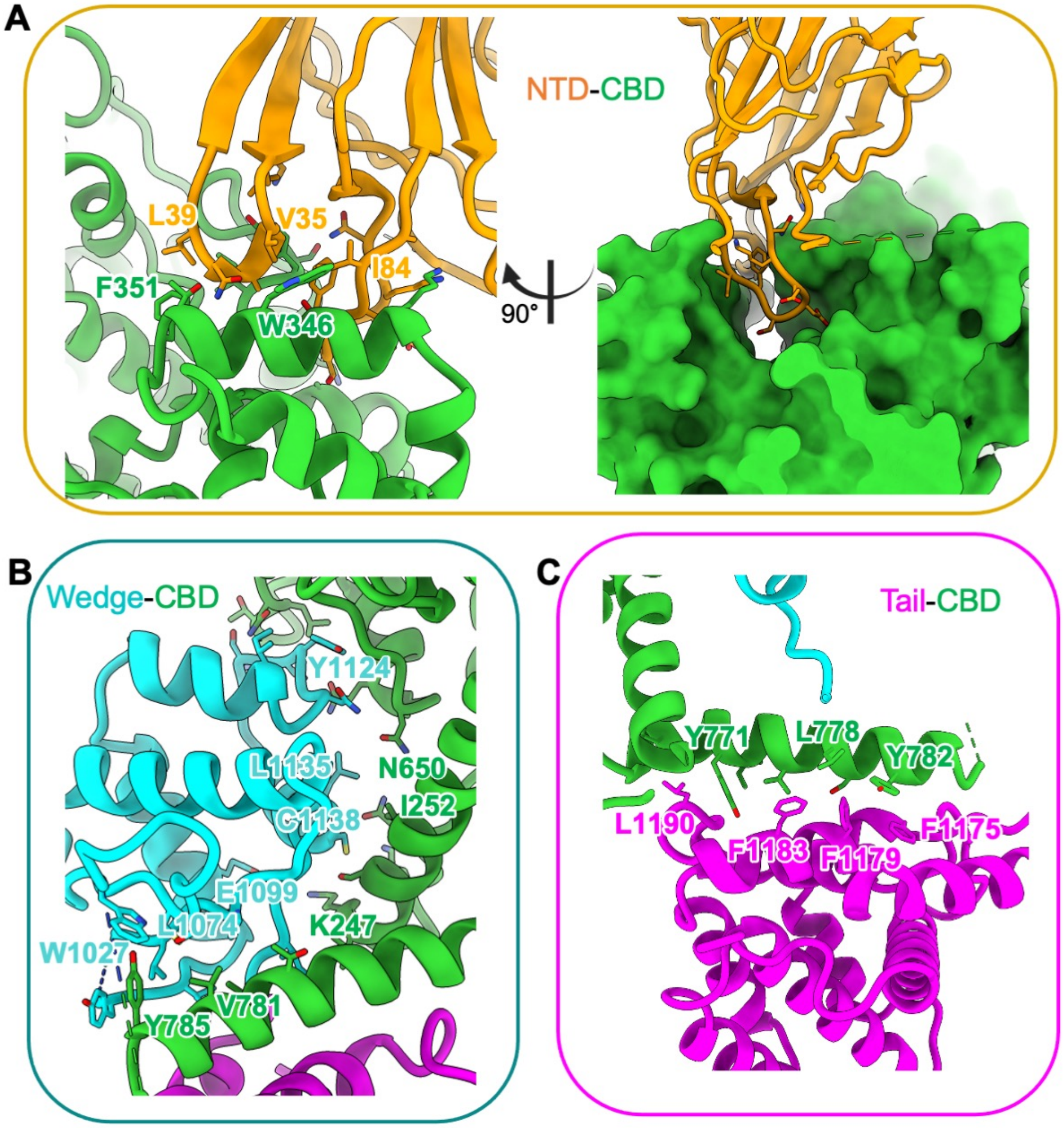

Supplementary Fig. S2. Interdomain interactions within PdpC*.* (A) Two different views illustrating the interactions between NTD (gold) and CBD (green), with key residues shown as sticks and labeled. **(B)** Interactions between Wedge (cyan) and CBD (green). **(C)** Interactions between Tail (magenta) and CBD (green) are mediated by two α-helices.

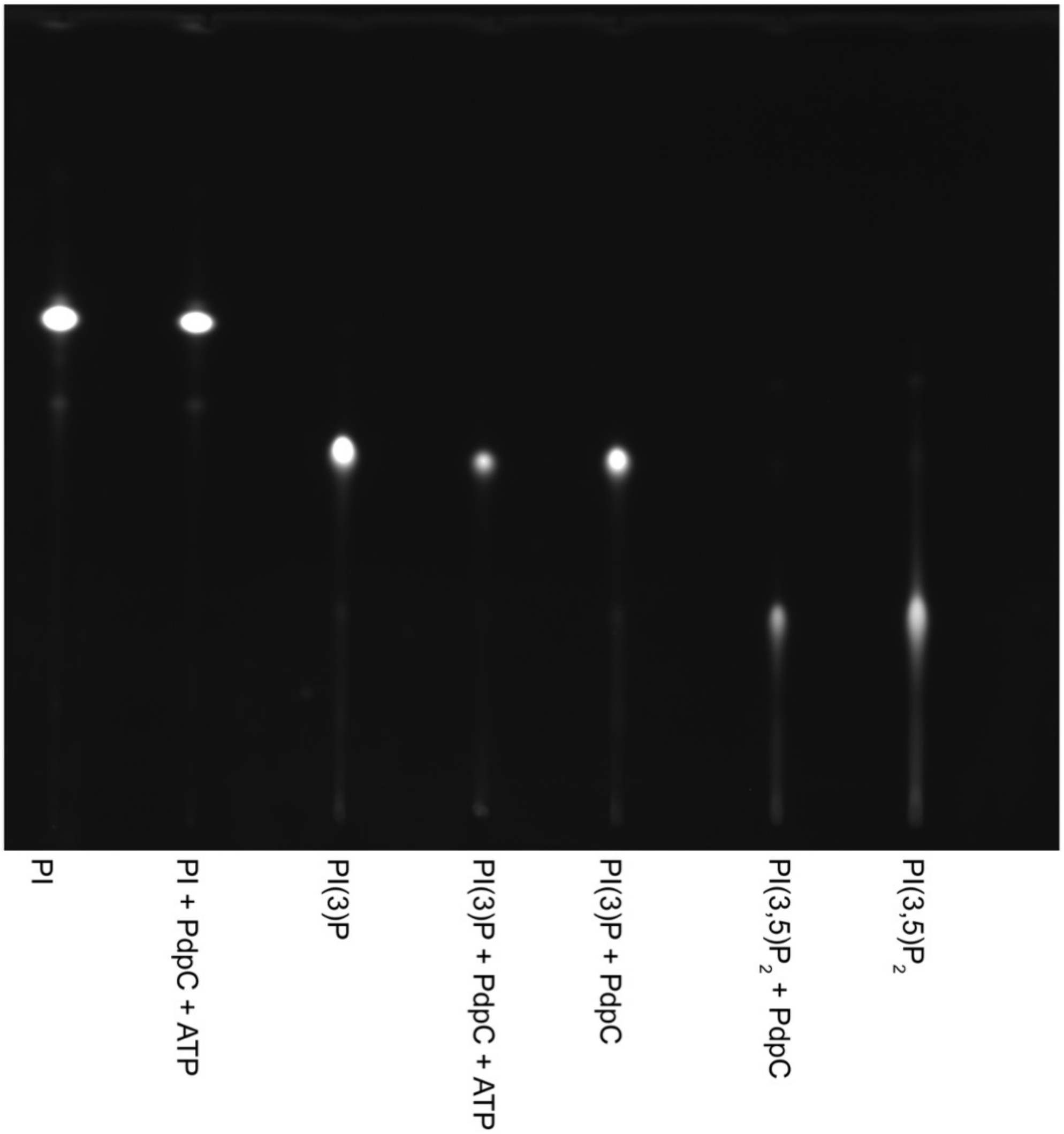

Supplementary Fig. S3: Phosphoinositide kinase and phosphatase activity are not detected in PdpC. PdpC was incubated with 50 μM BODIPY-labeled PI, PI(3)P, or PI(3,5)P_2, with or without 10 mM ATP and 10 mM $MgCl_2$ for 90 min at 37°C. The reaction was stopped by addition of acetone, dried under vacuum, and BODIPY-labeled lipids extracted with $CHCl_3$, spotted onto a silica thin layer chromatography plate, developed in $CHCl_3$:MeOH:Acetone:HOAc:Water (70:50:20:20:20), and imaged under blue light. Equal amounts of standard PI, PI(3)P, and PI(3,5)P_2 that were incubated in the same buffer conditions without PdpC are included as reference controls.

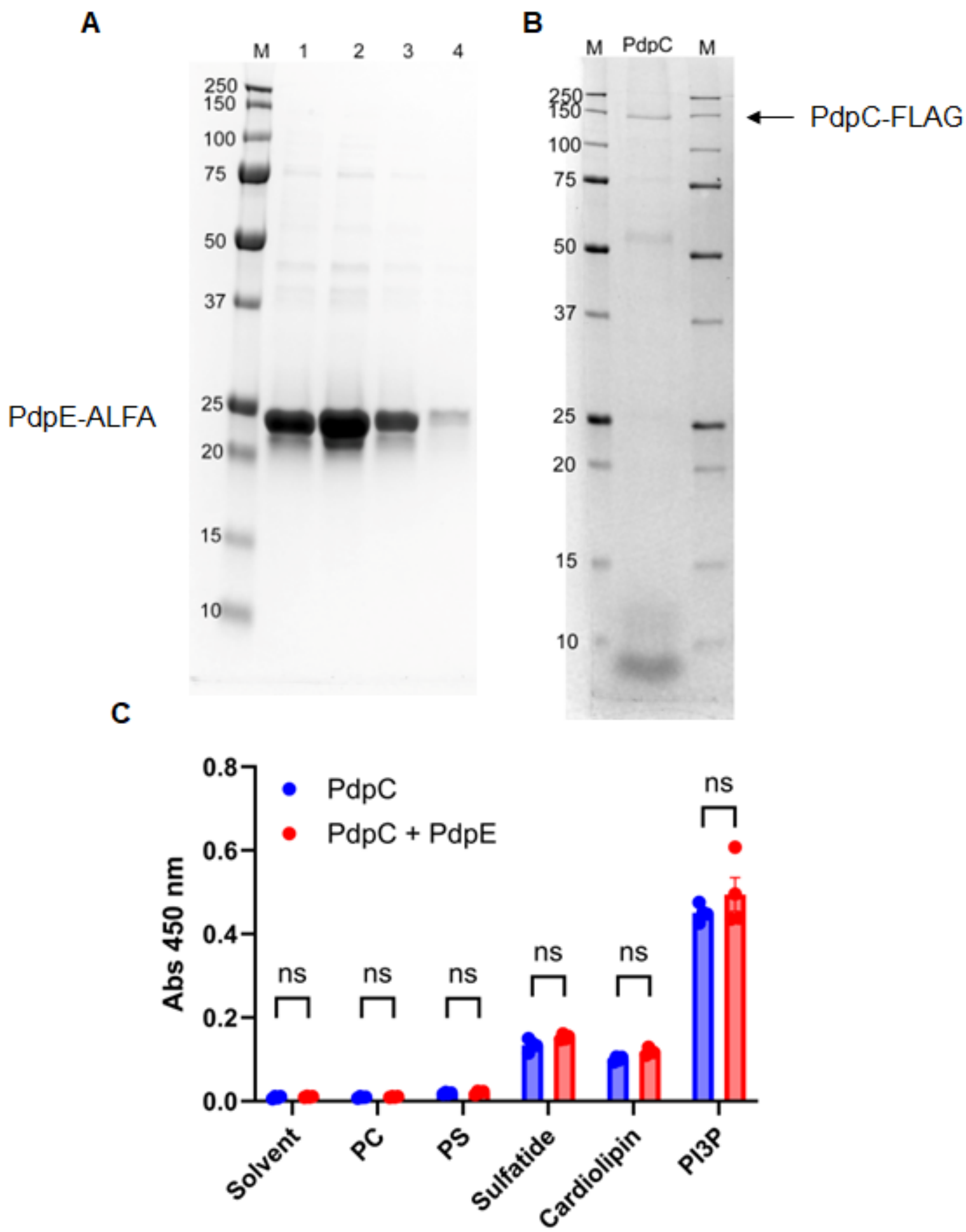

Supplementary Fig. S4: Pre-incubation of PdpC with PdpE does not alter PdpC lipid binding. (**A**) Coomassie blue stained SDS-PAGE gel showing purification of PdpE-ALFA by affinity chromatography using ALFA-selector Cold Elutable resin. Lanes 1 – 4 are consecutive elutions of the resin with ALFA peptide. M, molecular weight markers. (**B**) Coomassie blue stained SDS-PAGE gel showing PdpC-FLAG purified by FLAG-peptide elution from anti-FLAG affinity resin. (**C**) Purified PdpC-FLAG (1 µg/mL, shown in panel B), with or without pre-incubation with a 360-fold molar excess of PdpE-ALFA for 1 hour at room temperature, was added to wells of an ELISA plate coated with the indicated lipid or vehicle solvent. Lipid binding was detected by HRP-conjugated mouse anti-FLAG antibody and tetramethylbenzidine peroxidase substrate. PC, phosphatidylcholine; PS, phosphatidylserine. Data shown are means and standard errors of 4 replicates. Bars with “ns” indicate that analysis by ANOVA found no significant difference in lipid binding activity between the indicated conditions.

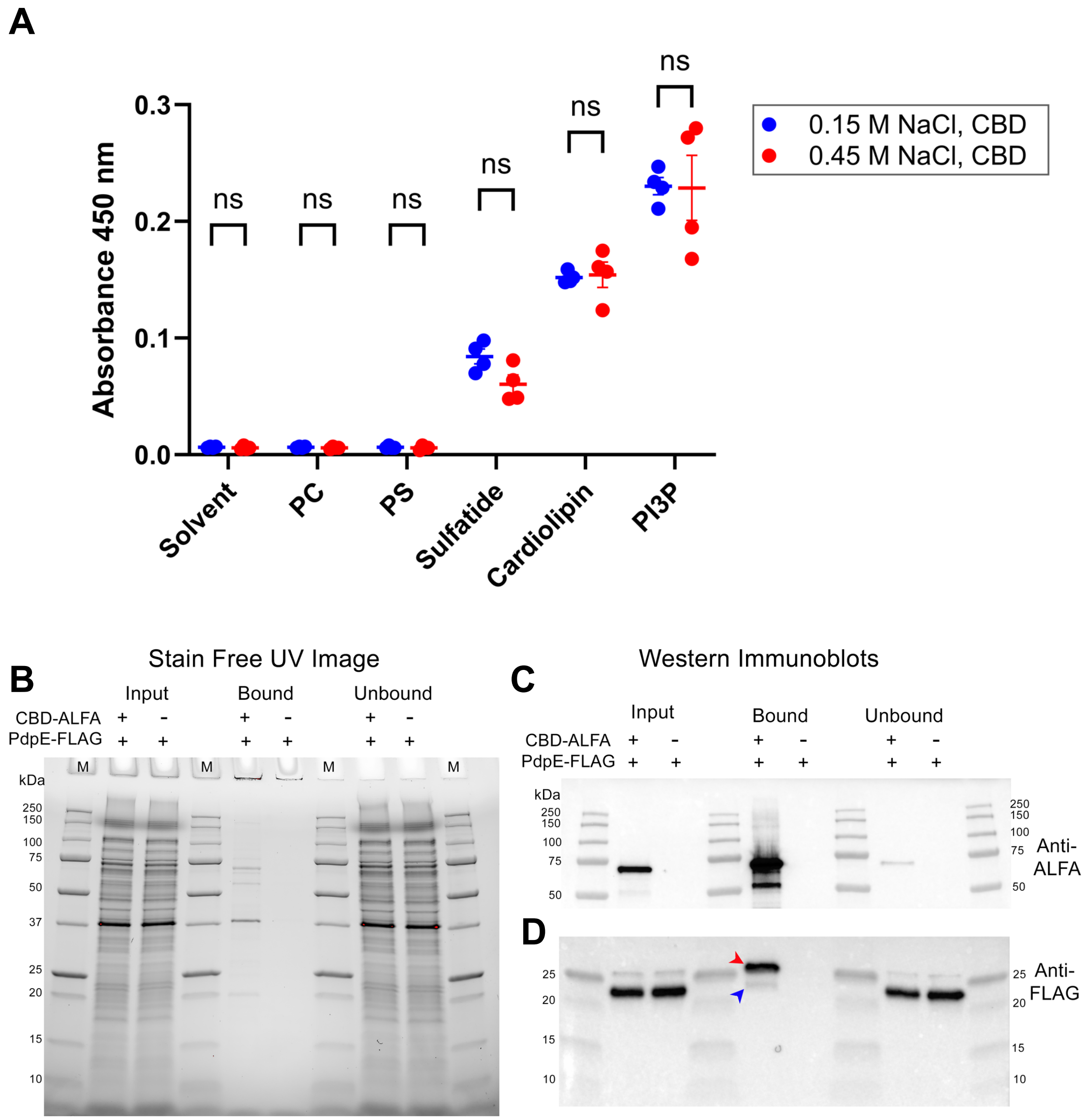

Supplementary Fig. S5: The CBD of PdpC binds to PI(3)P, cardiolipin and PdpE. (A) PI(3)P and cardiolipin binding. Lysates of *F. novicida* producing CBD-ALFA were used to probe ELISA plate wells coated with phosphatidylcholine (PC), phosphatidylserine (PS), sulfatide, cardiolipin (CL), or PI(3)P in Tris buffer containing 1% BSA, 0.1% TX-100, and either 0.15 M or 0.45 M NaCl. Binding was detected using HRP-conjugated and-ALFA nanobody and peroxidase substrate. Data indicate means and standard errors of four determinations. Bars with "ns" indicate that analysis by ANOVA found no significant difference between the 0.15 M and 0.45 M NaCl conditions. **(B-D)** PdpE binding. Lysates of *F. novicida* producing both PdpE-FLAG and CBD-ALFA or only PdpE-FLAG were incubated with ALFA-select resin to pull down CBD-ALFA. Input samples, proteins pulled down by anti-ALFA resin, and unbound proteins were analyzed by Stain free UV imaging (**B**) and by Western Immunoblotting for CBD-ALFA (**C**) and PdpE-FLAG (**D**). Sizes of molecular markers (M) are indicated in kDa. The red and blue arrowheads in Panel **D** indicate immunoreactive bands of PdpE-FLAG corresponding to unprocessed and processed PdpE-FLAG, respectively.

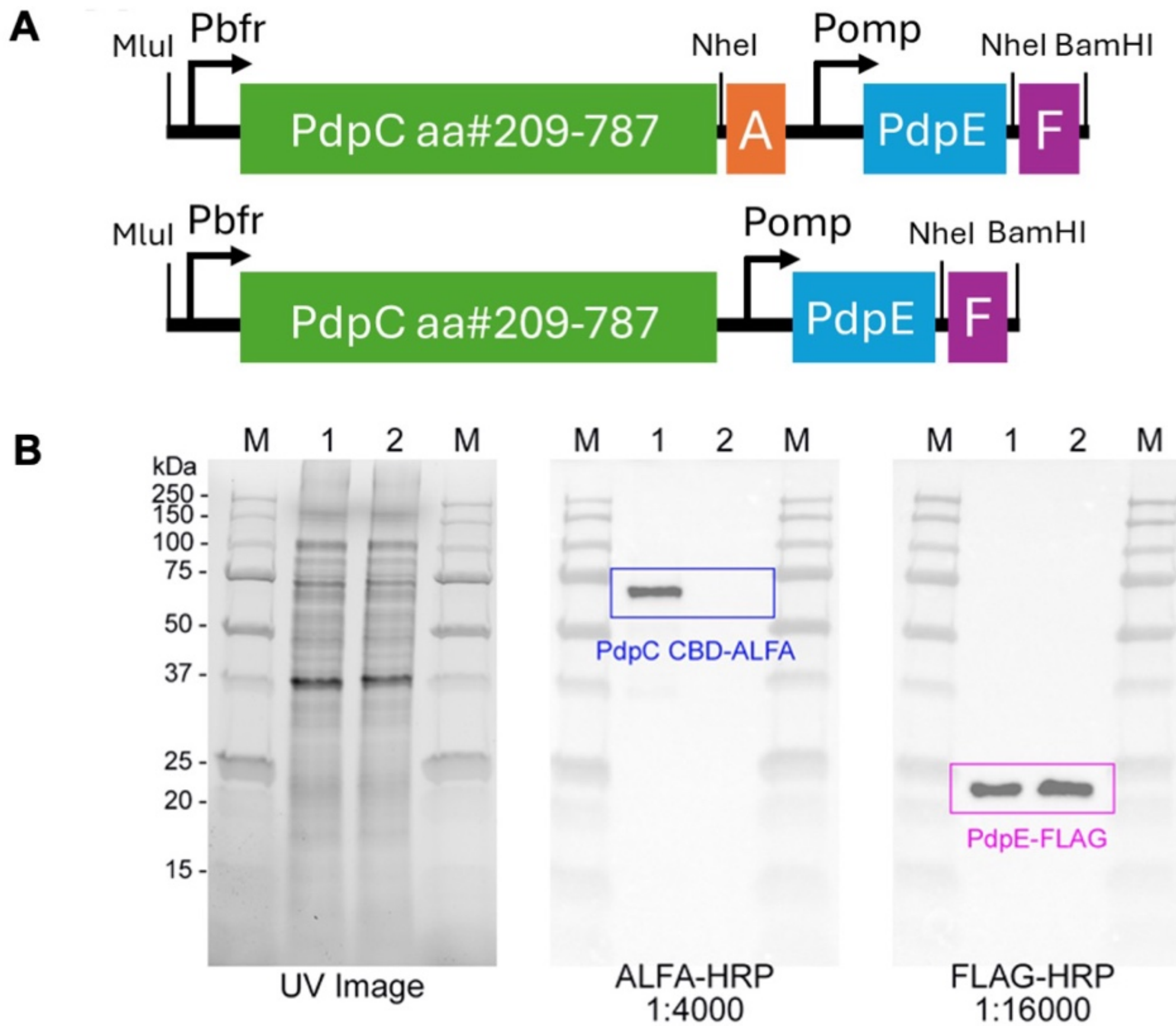

Supplementary Fig. S6: Co-production of epitope-tagged PdpC CBD and PdpE. (A) Bi-cistronic expression cassette containing coding sequence for PdpC central binding domain (CBD) with or without an ALFA tag and PdpE-FLAG cloned in pMP633. The plasmid constructs were transformed into *F. novicida* for use in the PdpC domain-based pulldown study. A, ALFA tag; F, FLAG tag. **(B)** Production of the epitope tagged PdpC CBD and PdpE was detected in the ultracentrifuge clarified bacterial lysates. Lane M, molecular weight markers; Lane 1, *F. novicida* carrying pMP633-pdpC-CBD-ALFA and pdpE-FLAG; Lane 2, *F. novicida* carrying pMP633-pdpC-CBD and pdpE-FLAG.

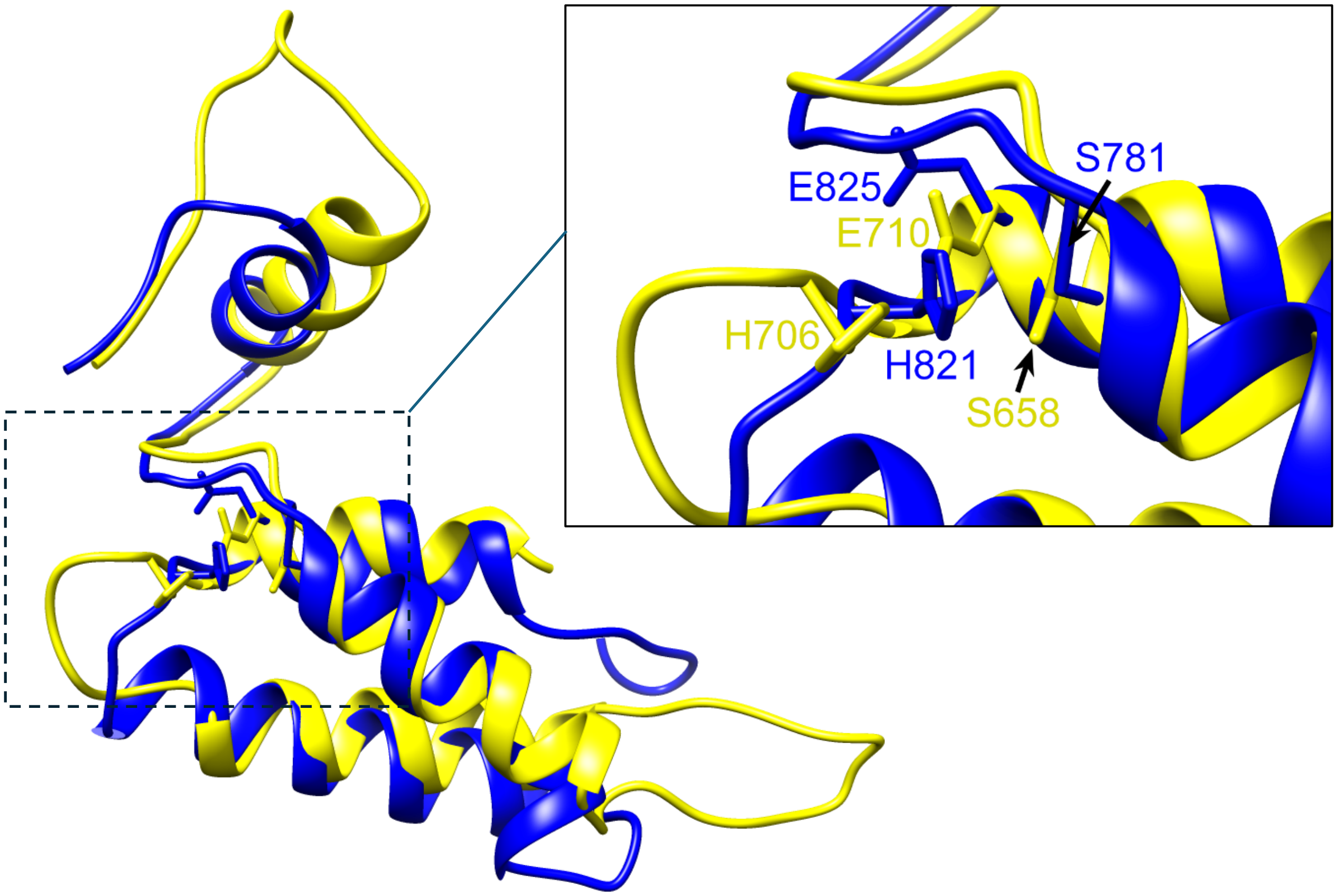

Supplementary Fig. S7. Alignment of MCF-SHE regions of PdpC and *Photorhabdus luminescens* Makes Caterpillars Floppy 1 Toxin (Mcf1). (A) Although lacking significant sequence homology, the MCF-SHE region of PdpC (residues 626 – 718, in yellow) shows structural homology with residues 758 – 840 of Mcf1 (in blue). The enlarged inset panel shows that the putative catalytic triad residues of PdpC (S658, H706, and E710) align well with the corresponding S781, H821, and E825 residues of Mcf1 toxin (pdb 8P52).

Uncropped gels and blots

Fig. 4A

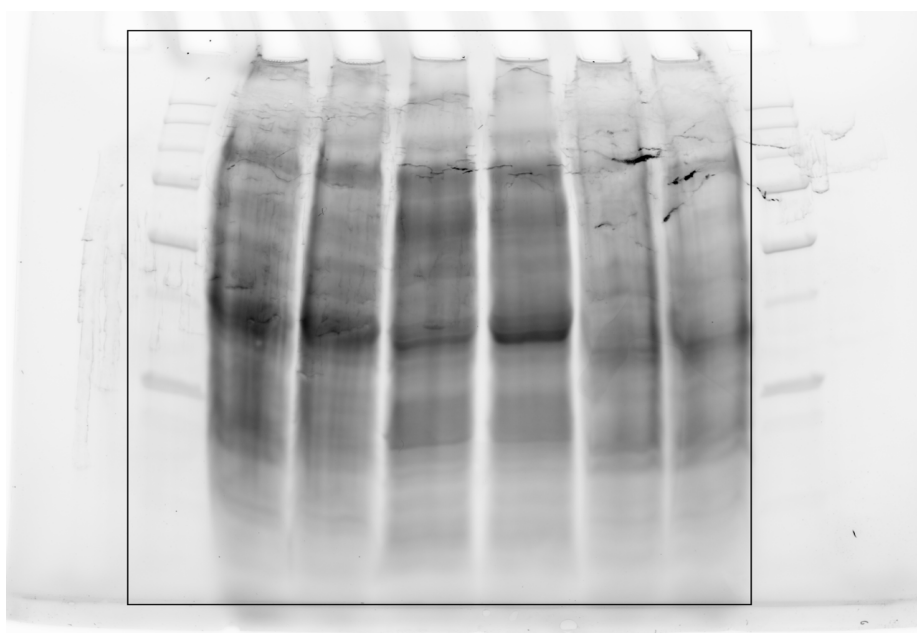

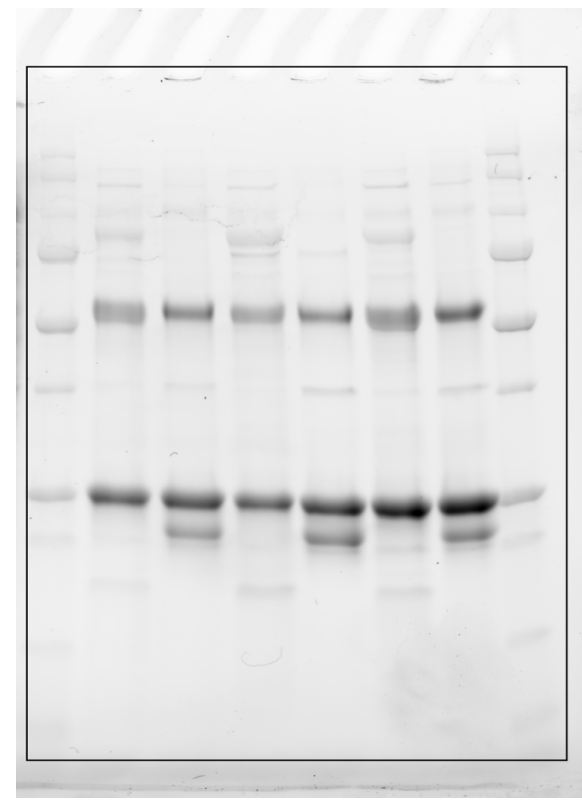

Fig. 4B

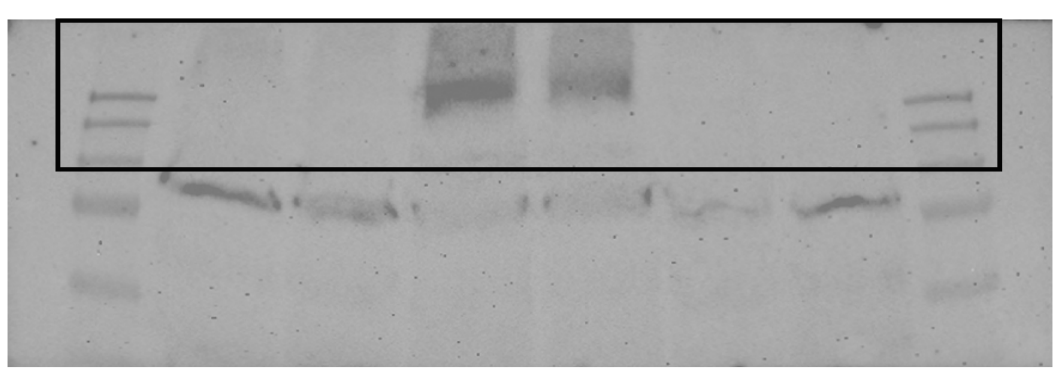

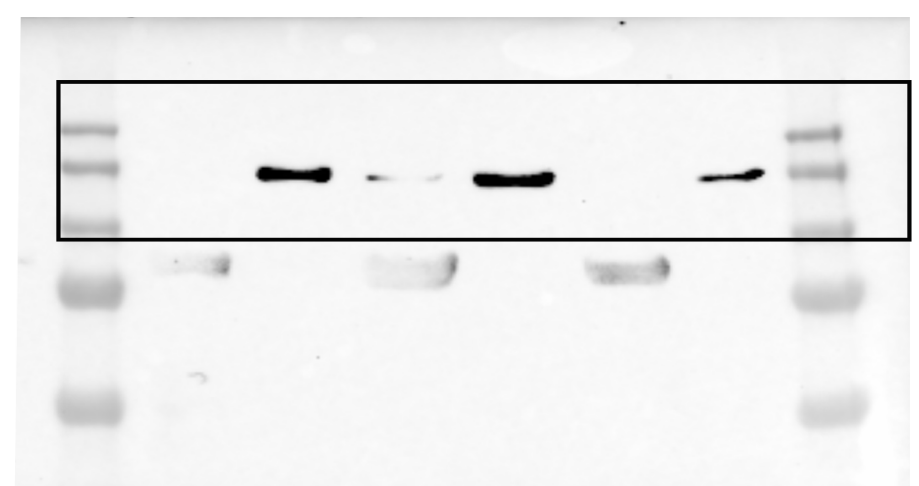

Fig. 4C

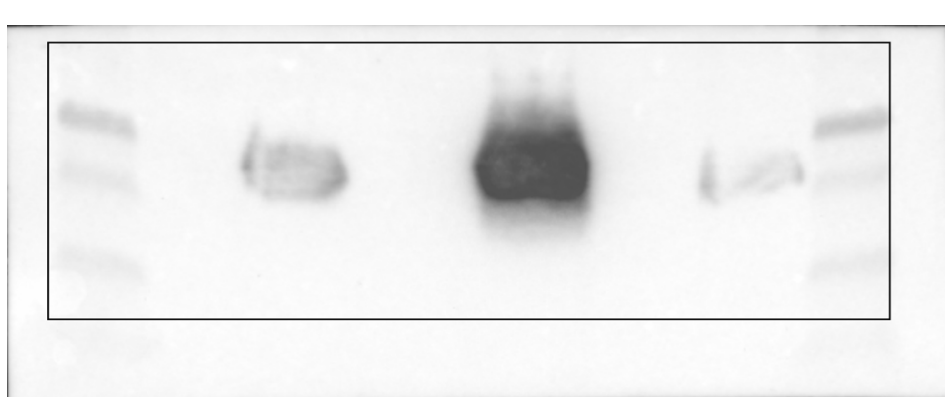

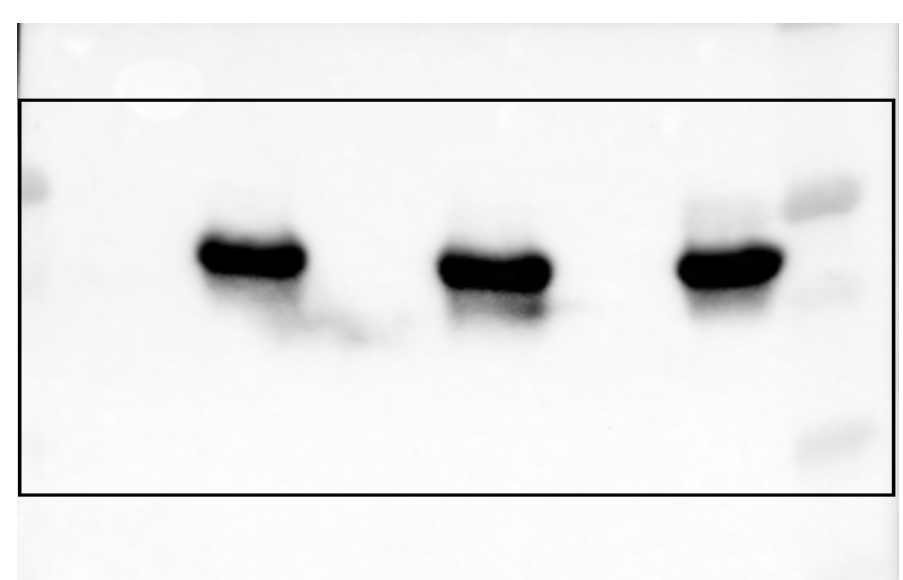

Fig. 4D

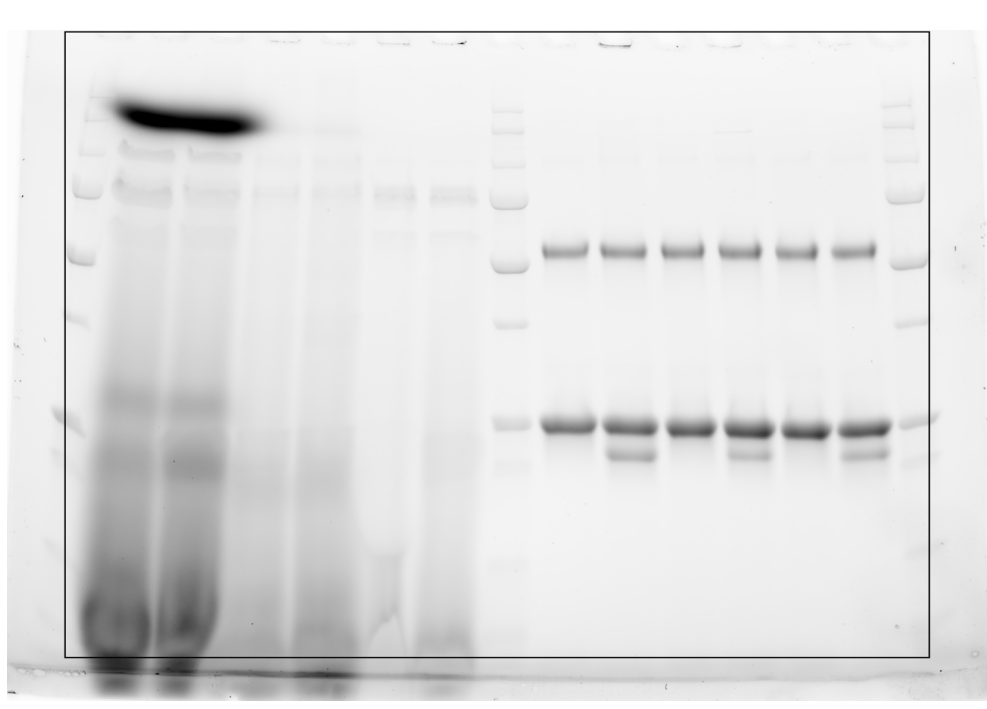

Fig. 4E

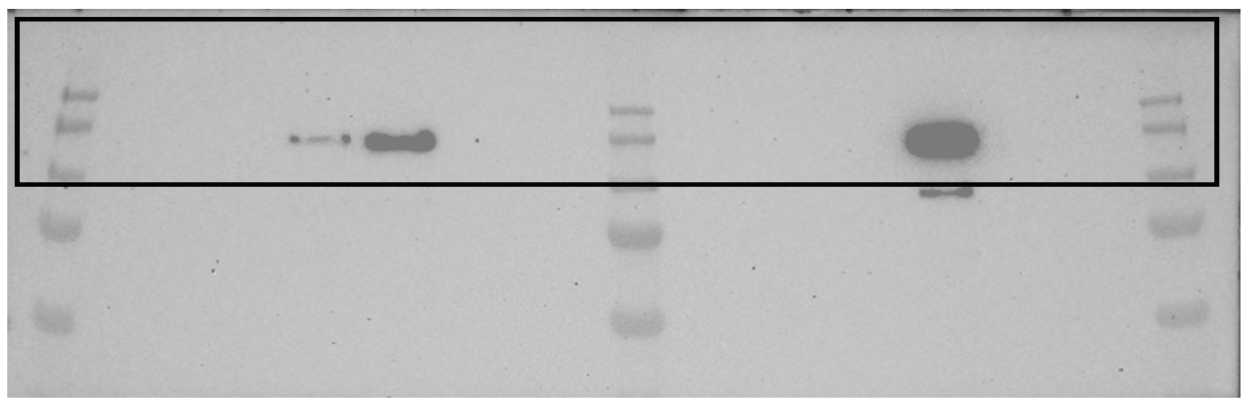

Fig. 4F

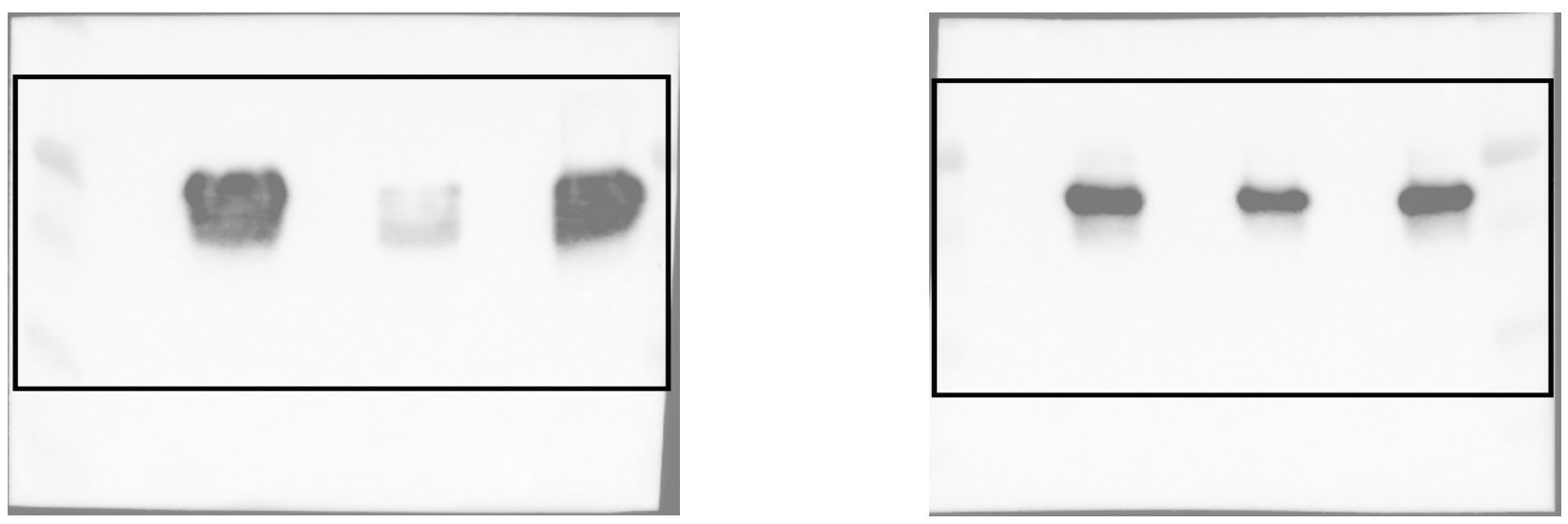

Fig. 4G

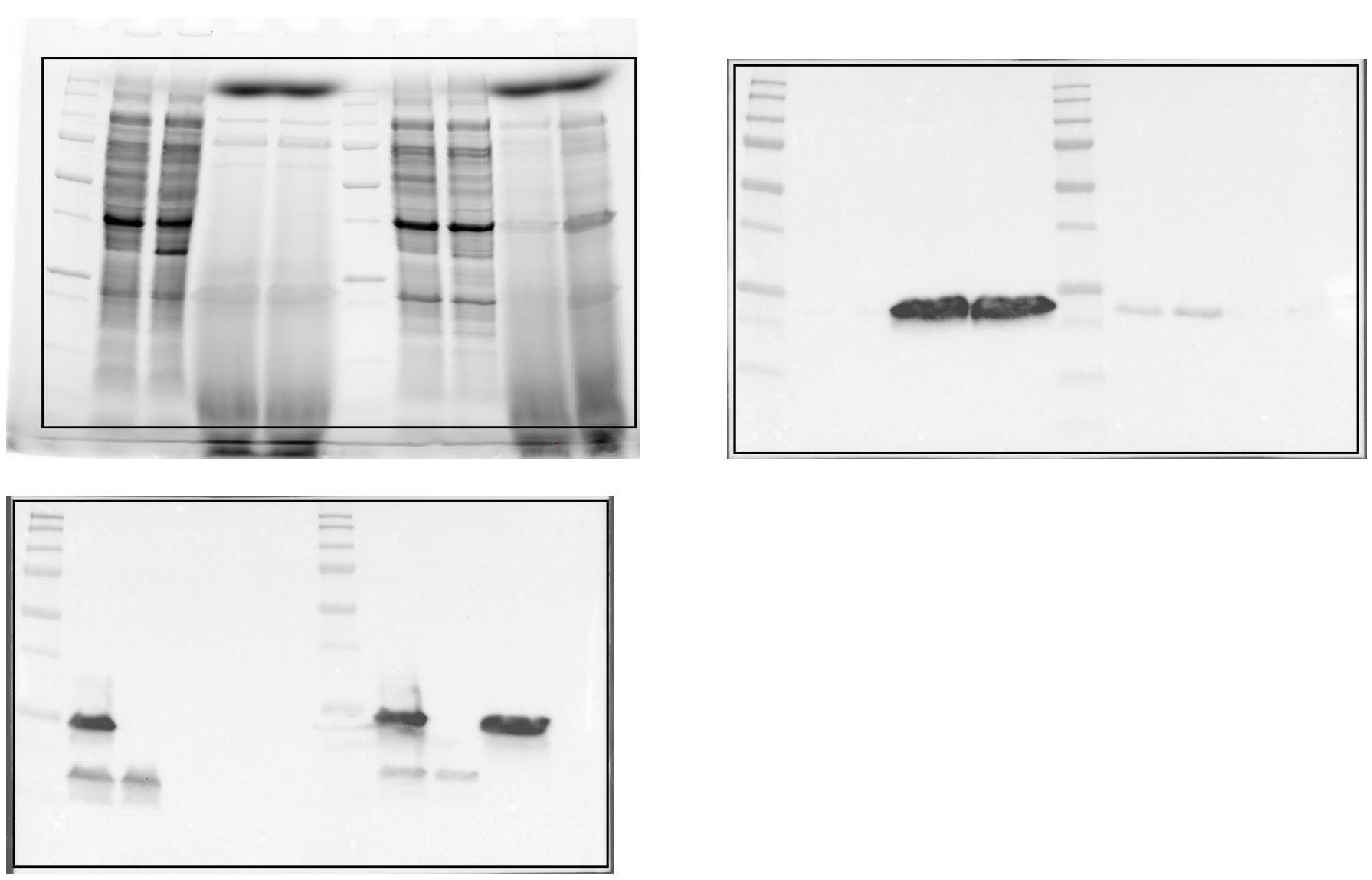

Fig. S4A

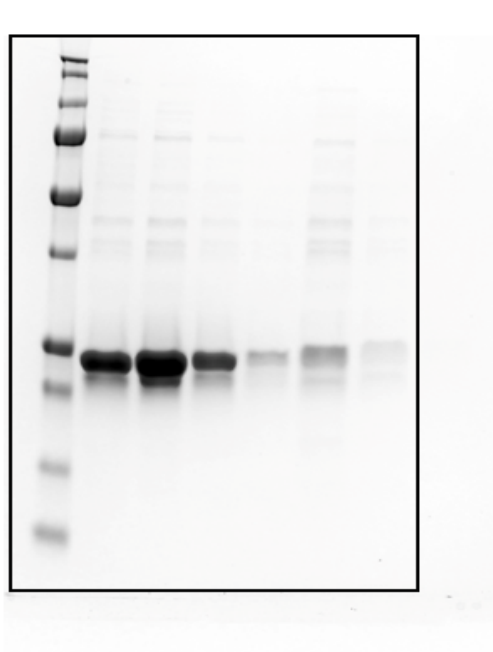

Fig. S4B

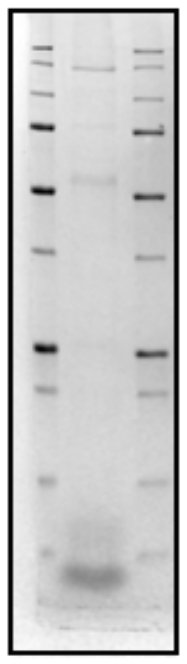

Fig. S5B

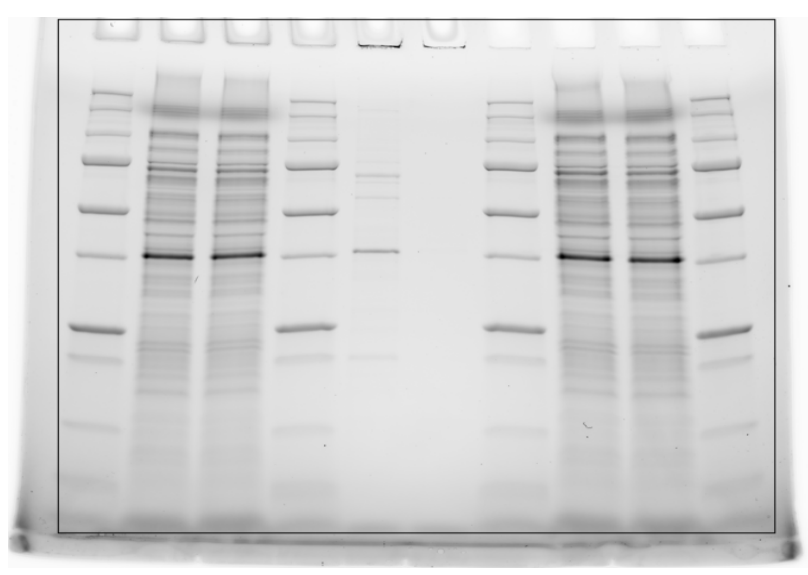

Fig. S5C

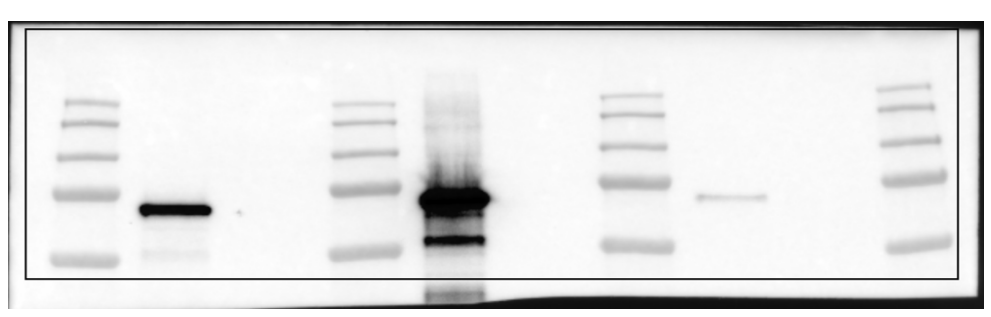

Fig. S5D

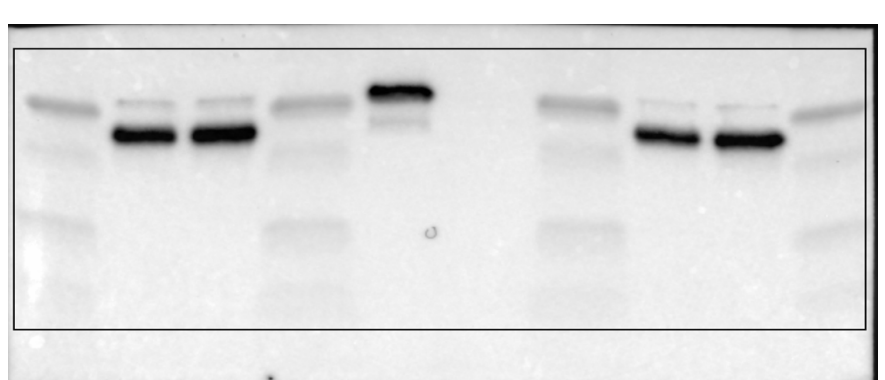

Fig. S6

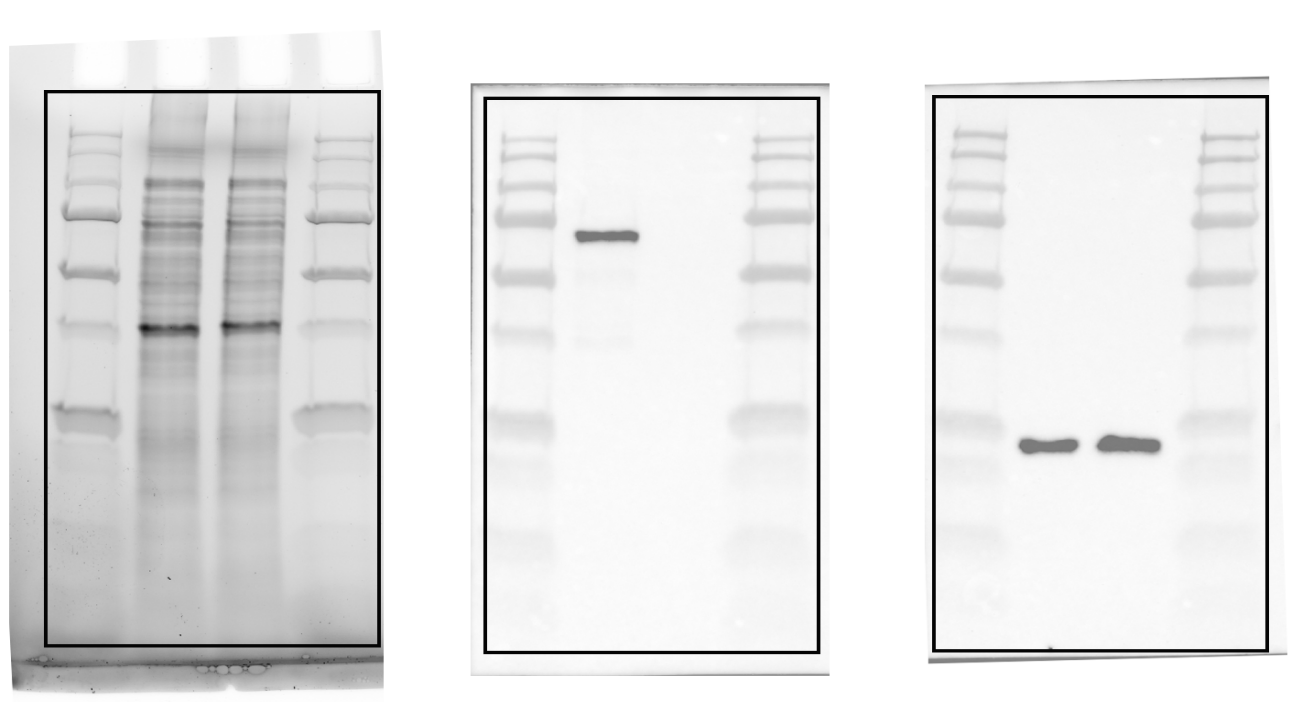

Supplementary Table 1. Raw ELISA absorbance (A450) values of PdpC binding to phospholipids in the presence or absence of PdpE

	Abs 450 nm							
	PdpC				PdpC + PdpE			
Solvent	0.008	0.006	0.011	0.011	0.01	0.009	0.012	0.011
PC	0.007	0.009	0.01	0.011	0.009	0.01	0.011	0.011
PS	0.017	0.018	0.02	0.021	0.016	0.024	0.021	0.023
Sulfatide	0.15	0.138	0.133	0.115	0.155	0.161	0.147	0.149
Cardiolipin	0.105	0.106	0.098	0.094	0.116	0.11	0.116	0.129
PI3P	0.476	0.452	0.426	0.448	0.438	0.496	0.436	0.608

Supplementary Table 2. Raw ELISA absorbance (A450) values for CBD binding to phospholipids at 0.15 M and 0.45 M NaCl

	**Absorbance 450 nm**							
	**0.15 M NaCl, CBD**				**0.45 M NaCl, CBD**			
**Solvent**	0.007	0.007	0.006	0.006	0.005	0.005	0.008	0.006
**Lecithin (PC)**	0.006	0.007	0.006	0.007	0.005	0.007	0.006	0.006
**Phosphatidy**	0.006	0.006	0.008	0.006	0.006	0.004	0.006	0.008
**Sulfatide**	0.091	0.078	0.098	0.07	0.049	0.048	0.064	0.081
**Cardiolipin**	0.149	0.148	0.159	0.152	0.157	0.161	0.175	0.124
**PI3P**	0.247	0.229	0.211	0.234	0.195	0.168	0.272	0.28
