# Supplementary material for: Structure and potential role of T6SS effector PdpC in Francisella tularensis intracellular lifestyle
Source: Commun Biol. 2026 Jun 25;9:1352. doi: 10.1038/s42003-026-10543-y (PMC13669386; doi:10.1038/s42003-026-10543-y)
Supplement: Supplementary file 2 [file 42003_2026_10543_MOESM2_ESM.pdf]

nature portfolio

Corresponding author(s): Z. Hong Zhou

Last updated by author(s): 04/10/2026

Reporting Summary

Nature Portfolio wishes to improve the reproducibility of the work that we publish. This form provides structure for consistency and transparency in reporting. For further information on Nature Portfolio policies, see our Editorial Policies and the Editorial Policy Checklist.

Statistics

For all statistical analyses, confirm that the following items are present in the figure legend, table legend, main text, or Methods section.

n/a	Confirmed	
☐	☒	The exact sample size (*n*) for each experimental group/condition, given as a discrete number and unit of measurement
☐	☒	A statement on whether measurements were taken from distinct samples or whether the same sample was measured repeatedly
☒	☐	The statistical test(s) used AND whether they are one- or two-sided<br>*Only common tests should be described solely by name; describe more complex techniques in the Methods section.*
☒	☐	A description of all covariates tested
☒	☐	A description of any assumptions or corrections, such as tests of normality and adjustment for multiple comparisons
☒	☐	A full description of the statistical parameters including central tendency (e.g. means) or other basic estimates (e.g. regression coefficient) AND variation (e.g. standard deviation) or associated estimates of uncertainty (e.g. confidence intervals)
☒	☐	For null hypothesis testing, the test statistic (e.g. *F*, *t*, *r*) with confidence intervals, effect sizes, degrees of freedom and *P* value noted<br>*Give P values as exact values whenever suitable.*
☒	☐	For Bayesian analysis, information on the choice of priors and Markov chain Monte Carlo settings
☒	☐	For hierarchical and complex designs, identification of the appropriate level for tests and full reporting of outcomes
☒	☐	Estimates of effect sizes (e.g. Cohen's *d*, Pearson's *r*), indicating how they were calculated

Our web collection on statistics for biologists contains articles on many of the points above.

Software and code

Policy information about availability of computer code

Data collection	SerialEM 4.0
Data analysis	Topaz v0.2.5, CryoSPARC, RELION3.1, RELION5.0, UCSF ChimeraX 1.25, Coot0.8.9, PHENIX 1.19

For manuscripts utilizing custom algorithms or software that are central to the research but not yet described in published literature, software must be made available to editors and reviewers. We strongly encourage code deposition in a community repository (e.g. GitHub). See the Nature Portfolio guidelines for submitting code & software for further information.

Data

Policy information about availability of data

All manuscripts must include a data availability statement. This statement should provide the following information, where applicable:

- Accession codes, unique identifiers, or web links for publicly available datasets
- A description of any restrictions on data availability
- For clinical datasets or third party data, please ensure that the statement adheres to our policy

The cryoEM density map has been deposited in the Electron Microscopy Data Bank under accession code 9YNB. The atomic coordinate has been deposited in the Protein Data Bank under accession code EMD-73168.

Research involving human participants, their data, or biological material

Policy information about studies with human participants or human data. See also policy information about sex, gender (identity/presentation), and sexual orientation and race, ethnicity and racism.

Reporting on sex and gender	This study did not use human participant/data/material.
Reporting on race, ethnicity, or other socially relevant groupings	This study did not use human participant/data/material.
Population characteristics	This study did not use human participant/data/material.
Recruitment	This study did not use human participant/data/material.
Ethics oversight	This study did not use human participant/data/material.

Note that full information on the approval of the study protocol must also be provided in the manuscript.

Field-specific reporting

Please select the one below that is the best fit for your research. If you are not sure, read the appropriate sections before making your selection.

☒ Life sciences ☐ Behavioural & social sciences ☐ Ecological, evolutionary & environmental sciences

For a reference copy of the document with all sections, see nature.com/documents/nr-reporting-summary-flat.pdf

Life sciences study design

All studies must disclose on these points even when the disclosure is negative.

Sample size	No statistical methods were used to predetermine sample size. Cryo-EM 3D reconstructions were calculated from 39482 images. The data size is comparable to other studies in this field. The number of particles used for each final map is sufficient to obtain reliable classification and reconstruction results by cryo-EM.
Data exclusions	For cryo-EM analysis, particles that do not belong to the class of interest or have poor qualities based on well established cryo-EM principle were excluded after rounds of 2D and 3D classification. This is standard practice required to obtain high resolution cryo-EM structure of the class of interest.
Replication	For biochemical analysis, all results were independently replicated at least 3 times.
Randomization	For cryo-EM, datasets were randomly split into two halves for refinements and cross correlation coefficients (FSC curves) between the two half sets are calculated.
Blinding	For cryo-EM, datasets were automatically split into two halves in Relion, blinding is not feasible.

Reporting for specific materials, systems and methods

We require information from authors about some types of materials, experimental systems and methods used in many studies. Here, indicate whether each material, system or method listed is relevant to your study. If you are not sure if a list item applies to your research, read the appropriate section before selecting a response.

Materials & experimental systems

n/a	Involved in the study	
☐	☒	Antibodies
☐	☒	Eukaryotic cell lines
☒	☐	Palaeontology and archaeology
☒	☐	Animals and other organisms
☒	☐	Clinical data
☒	☐	Dual use research of concern
☒	☐	Plants

Methods

n/a	Involved in the study	
☒	☐	ChIP-seq
☒	☐	Flow cytometry
☒	☐	MRI-based neuroimaging

Antibodies

Antibodies used	HRP-conjugated M2 anti-FLAG antibody (Sigma Aldrich), polyclonal rabbit anti-PdpC antibody (BEI Resourcs) , HRP conjugated goat anti-rabbit IgG antibody (BioRad)
Validation	These antibodies are directly purchased from vendors listed above.

Eukaryotic cell lines

Policy information about cell lines and Sex and Gender in Research

Cell line source(s)	HeLa Tet-off cells
Authentication	HeLa Tet-off cell was not authenticated since it was directly purchased
Mycoplasma contamination	Cell line was tested negative for Mycoplasma contamination
Commonly misidentified lines (See ICLAC register)	This cell line is not listed in ICLAC as commonly misidentified cell line.

Plants

Seed stocks	Plants were not used in this study.
Novel plant genotypes	Plants were not used in this study.
Authentication	Plants were not used in this study.
